# Multidimensional associations between nutrient intake and healthy ageing in humans

**DOI:** 10.1186/s12915-022-01395-z

**Published:** 2022-09-01

**Authors:** Alistair M. Senior, Véronique Legault, Francis B. Lavoie, Nancy Presse, Pierrette Gaudreau, Valérie Turcot, David Raubenheimer, David G. Le Couteur, Stephen J. Simpson, Alan A. Cohen

**Affiliations:** 1 University of Sydney, Charles Perkins Centre, Camperdown, New South Wales 2006 Australia; 2 University of Sydney, School of Life and Environmental Science, Camperdown, New South Wales 2006 Australia; 3 University of Sydney, School of Mathematics and Statistics, Camperdown, New South Wales 2006 Australia; 4 Department of Family Medicine, Groupe de recherche PRIMUS, University of Sherbrooke, Sherbrooke, QC Canada; 5 Research Center on Aging, CIUSSS-de-l’Estrie-CHUS, Sherbrooke, QC Canada; 6 Centre de Recherche de l’Institut Universitaire de Gériatrie de Montréal, Montréal, QC Canada; 7 Department of Community Health Sciences, Faculty of Medicine and Health Sciences, Université de Sherbrooke, Sherbrooke, QC Canada; 8 Department of Medicine, Université de Montréal, Montréal, QC Canada; 9 Centre de recherche du centre hospitalier de l’Université de Montréal, Montréal, QC Canada; 10 University of Sydney, School of Medicine, Camperdown, New South Wales 2006 Australia; 11 Ageing and Alzheimers Institute and ANZAC Research Institute, Concord Hospital, Concord, New South Wales 2139 Australia; 12 Centre de recherche du centre hospitalier Universitaire de Sherbrooke, Sherbrooke, QC Canada; 13 Butler Columbia Aging Center, Mailman School of Public Health, Columbia University, New York, NY USA; 14 Department of Environmental Health Sciences, Mailman School of Public Health, Columbia University, New York, NY USA

**Keywords:** Ageing, Dysregulation, Geometric framework, Healthspan, Nutrition, Systems epidemiology

## Abstract

**Background:**

Little is known about how normal variation in dietary patterns in humans affects the ageing process. To date, most analyses of the problem have used a unidimensional paradigm, being concerned with the effects of a single nutrient on a single outcome. Perhaps then, our ability to understand the problem has been complicated by the fact that both nutrition and the physiology of ageing are highly complex and multidimensional, involving a high number of functional interactions. Here we apply the multidimensional geometric framework for nutrition to data on biological ageing from 1560 older adults followed over four years to assess on a large-scale how nutrient intake associates with the ageing process.

**Results:**

Ageing and age-related loss of homeostasis (physiological dysregulation) were quantified via the integration of blood biomarkers. The effects of diet were modelled using the geometric framework for nutrition, applied to macronutrients and 19 micronutrients/nutrient subclasses. We observed four broad patterns: (1) The optimal level of nutrient intake was dependent on the ageing metric used. Elevated protein intake improved/depressed some ageing parameters, whereas elevated carbohydrate levels improved/depressed others; (2) There were non-linearities where intermediate levels of nutrients performed well for many outcomes (i.e. arguing against a simple more/less is better perspective); (3) There is broad tolerance for nutrient intake patterns that don’t deviate too much from norms (‘homeostatic plateaus’). (4) Optimal levels of one nutrient often depend on levels of another (e.g. vitamin E and vitamin C). Simpler linear/univariate analytical approaches are insufficient to capture such associations. We present an interactive tool to explore the results in the high-dimensional nutritional space.

**Conclusion:**

Using multidimensional modelling techniques to test the effects of nutrient intake on physiological dysregulation in an aged population, we identified key patterns of specific nutrients associated with minimal biological ageing. Our approach presents a roadmap for future studies to explore the full complexity of the nutrition-ageing landscape.

**Supplementary Information:**

The online version contains supplementary material available at 10.1186/s12915-022-01395-z.

## Background

How does what we eat affect our healthspan and longevity? The answer to this relatively concise question is unavoidably complex. Conventional approaches to understanding the effects of diet on health and ageing, particularly in human nutrition, have usually focussed on single nutrients or a handful of dietary attributes/patterns [1–3]. Yet, nutrients have both individual and interactive effects. For example, at the most macro-level, protein, carbohydrate and fat energy sources interact to determine metabolic, physiological and cognitive functioning (e.g. [4–6]). Similarly, the numerous phenotypic changes that occur with age are increasingly recognised as interconnected and multidimensional [7–10]. Thus seemingly distinct physiological components of ageing likely reflect a broader loss of homeostasis in a complex dynamic system rather than independent processes [11]. Such interdependencies mean that the atomised interpretation of the effects of a single nutrient, diet, molecular mechanism or biomarker is likely to be context-dependent [12–15]; a consequence being that the results of univariate studies are spurious and/or more difficult to reproduce, leading to inconsistent conclusions between studies.

**Fig. 1:**
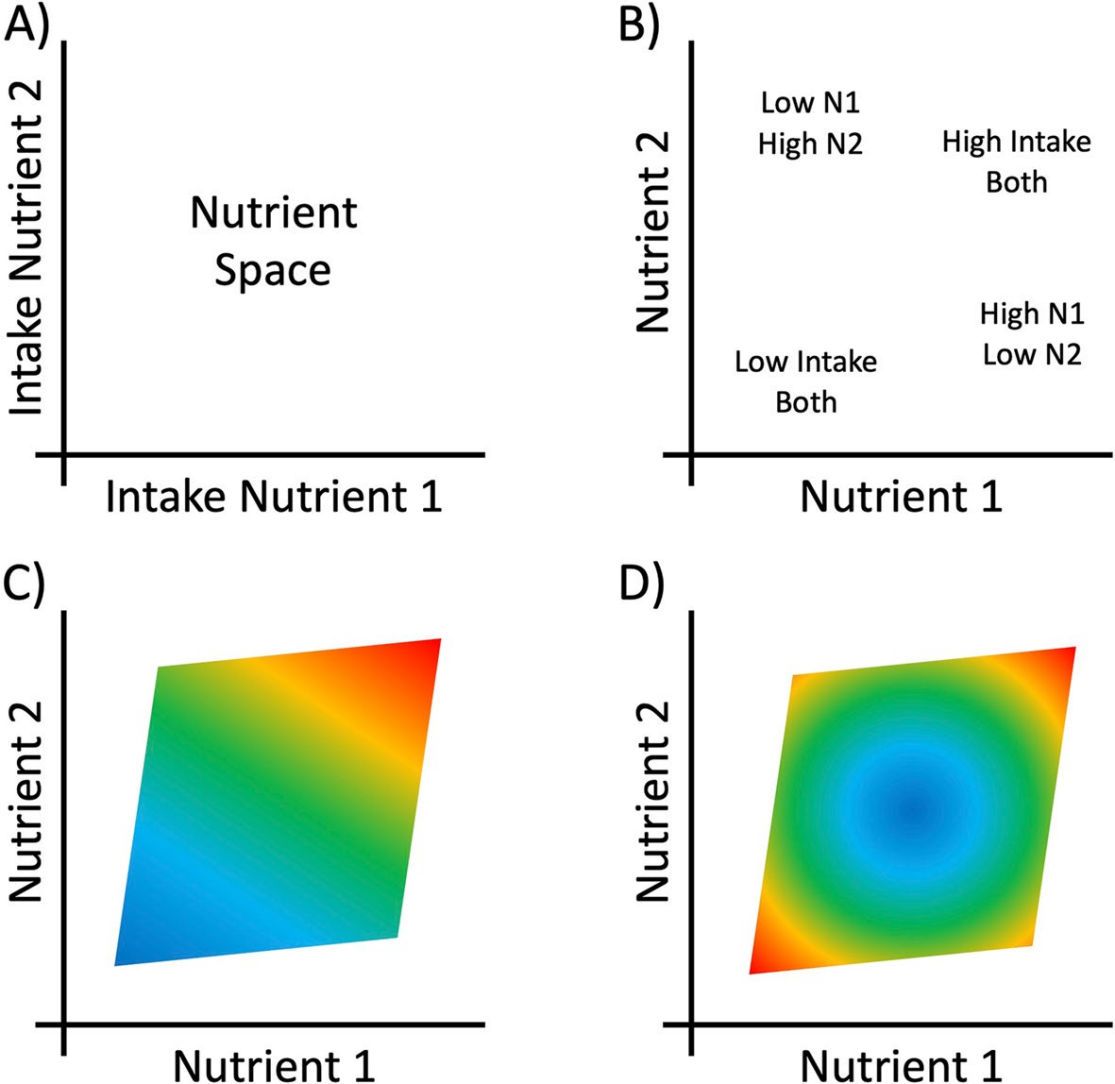
The geometric framework for nutrition (GFN) provides a multi-dimensional perspective on nutrition, by considering the intake of multiple nutrients simultaneously. **A** A 2-dimensional nutrient space with intake of nutrient 1 on the *x*-axis and intake of nutrient 2 on the *y*-axis. **B** Each point within the nutrient space represents some level of intake of the two nutrients. The effects of the two nutrients on an outcome of interest can be estimated using a statistical model fitted from data gathered on intake of the nutrients and the outcome. Predictions from the model can then be shown as a coloured topology surface overlaid on the nutrient space. Here generalised additive models are used to look at nutrient intake and ageing/dysregulation in a cohort of people aged 67+. **C** An example surface showing a linear additive effect of intake of both nutrients on the outcome, where low intakes lead to low-value outcome (blue colour) and high intakes lead to a high value of the outcome (red colour). **D** An example surface showing a non-linear effect of intake of both nutrients on the outcome, where moderate intakes lead to low-value outcomes. These surfaces could be adjusted for other factors (e.g. age) by including covariates in the statistical model

The Geometric Framework for Nutrition (GFN) is a state-space approach to nutrition that deals with dietary complexity by considering multiple dimensions of nutrient intake simultaneously (Fig. 1) [16, 17]. Using fully factorial experiments, the GFN has shown that components of biological ageing are affected by the ratio of dietary macronutrients independently of the effects of net energy intake. These effects have been observed across taxa [18–20]. For example, mice subjected to a lifetime of low-protein (within the boundaries of what can support growth and development) high-carbohydrate intake display improved cardiometabolic health and increased median age at death relative to animals with higher protein or fat intakes [4, 21]. Any such benefits seemingly disappear in old age, though, when higher protein reduces late-life mortality [21]. These experimental findings in mice are consistent with epidemiological studies in humans that emphasise the importance of dietary protein for the elderly (reviewed in [22, 23]). Ecological analyses of national-level data in humans have also found that even at this highest order level, the macronutrient composition of the food supply is a powerful predictor of international variation in patterns of mortality [24]. Collectively, these studies emphasise the importance of multi-dimensional thinking in nutrition.

The biological ageing process is no more tractable than nutrition. There is no clear consensus as to what ageing is [25], though most researchers now agree it is multi-factorial [26, 27]. Different methods for quantifying ageing correlate poorly with one another after chronological age adjustment [28], implying that ageing is a compound process. An emerging approach is to measure the effects of ageing via the breakdown in homeostatic regulation (i.e. dysregulation) across physiological systems [10, 29], an approach complementary to ‘biological age’. A statistical distance can be used to quantify how abnormal, or ‘dysregulated’, an individual’s biomarker profile is, either globally or within specific systems. In this way, it is possible to generate dysregulation scores that are predictive of a wide array of health outcomes during ageing, including mortality, frailty, and chronic diseases [10, 30–33]. Dysregulation scores thus simultaneously provide a proxy for the ageing process and general health state. In this sense, dysregulation may be a better metric of health than measures of the ageing process per se, if such a thing actually exists.

Here, we apply the GFN to model the effects of nutrient intake on dysregulation and ageing scores in community-dwelling older adults (>67 years old). We use data from the Quebec Longitudinal Study on Nutrition and Successful Aging (NuAge) [34], which is extensive enough to permit the application of the GFN in an epidemiological context. We show that combining these two methods provides a means to integrate the complexity inherent to nutrition and ageing physiology. We begin by testing the hypothesis, suggested by the mouse data, that higher protein intakes during old age are associated with markers of improved health. Simultaneously, we also test whether protein interacts with the intake of the other macronutrients, notably whether an increase in the ratio of carbohydrate to protein reduces markers of ageing. We then show how the same approach can be used inductively to detect micronutrient interactions that have systemic effects.

## Results

### Overview

Briefly, NuAge participants were community-dwelling men and women, aged 67-84 years in the Montreal, Laval, or Sherbrooke areas in Quebec (Canada). They were selected randomly from the Quebec Medicare database (*n*=36,183), after stratification for age and sex. Individuals with good general health were recruited (*n*=1793) between November 2003 and June 2005 (T1) [34]. Participants were re-examined annually for 3 years (T2, T3 and T4). Dietary intake data were collected annually using 3 non-consecutive 24-h diet recalls. Of the original recruits, 1754 (98%) provided consent for the integration of their data and biological specimens into the NuAge Database and Biobank for future studies. Measured from serum/plasma samples, 30 biomarkers were used to calculate dysregulation globally and for five systems that a previous study validated as being largely independent [10]: (1) oxygen transport; (2) liver/kidney function; (3) leukopoiesis; (4) micronutrients; and (5) lipids (see Table 1 for biomarkers in each score). We also calculated two other integrative, clinical-biomarker-based measures of biological ageing: phenotypic age (PhenoAge) and Klemera-Doubal biological age [35–37].

**Table 1:** Biomarkers used to calculate each dysregulation score, and their population mean ± standard deviation (SD)

Biomarker	System	Mean ± SD
Mean corpuscular haemoglobin, MCH (pg)	Oxygen transport	31.0 ± 1.6
MCH concentration (g/L)	Oxygen transport	340 ± 8
Mean corpuscular volume (fL)	Oxygen transport	91.3 ± 4.3
Red blood cell count (10^12^/L)	Oxygen transport	4.49 ± 0.43
Red cell distribution width^a^	Oxygen transport	0.136 ± 0.010
Haemoglobin (g/L)	Oxygen transport	139 ± 13
Albumin (g/L)	Liver/kidney function	43.2 ± 2.4
Creatinine (μmol/L)^a^	Liver/kidney function	80.3 ± 21.1
Albumin-globulin ratio	Liver/kidney function	1.52 ± 0.22
Bilirubin, total (μmol/L)^a^	Liver/kidney function	10.2 ± 4.3
Alkaline phosphatase (U/L)^a^	Liver/kidney function	78.3 ± 24.5
Alanine aminotransferase (U/L)^a^	Liver/kidney function	12.5 ± 22.5
Aspartate aminotransferase (U/L)^a^	Liver/kidney function	22.9 ± 22.8
γ-Glutamyltransferase (U/L)^a^	Liver/kidney function	33.0 ± 32.5
Lactate dehydrogenase (U/L)^a^	Liver/kidney function	158 ± 30
Proteins, total (g/L)	Liver/kidney function	71.9 ± 4.1
Uric acid (μmol/L)	Liver/kidney function	342 ± 82
Monocytes (differential count)	Leukopoiesis	0.076 ± 0.029
Neutrophils (differential count)	Leukopoiesis	0.616 ± 0.087
Leukocytes (10^9^/L)^a^	Leukopoiesis	6.36 ± 1.71
Lymphocytes (differential count)	Leukopoiesis	0.294 ± 0.081
Folate (nmol/L)^a^	Micronutrients	38.3 ± 12.5
Vitamin B12 (pmol/L)^a^	Micronutrients	431 ± 183
β-Carotene (μmol/L)^a^	Micronutrients	3.40 ± 5.55
α-Tocopherol (μmol/L^a^	Micronutrients	28.6 ± 16.0
γ-Tocopherol (μmol/L)^a^	Micronutrients	3.36 ± 2.72
Triglycerides (mmol/L)^a^	Lipids	1.63 ± 0.77
Cholesterol, total (mmol/L)	Lipids	5.12 ± 1.02
Cholesterol, high-density (mmol/L)	Lipids	1.44 ± 0.39
Platelet count (10^9^/L)^a^	-	237 ± 64

^a^ Variable was log-transformed to meet assumptions of normality

We assessed the effects of intakes of macronutrients and micronutrients, as well as their interactions, on measures of dysregulation and ageing. Our primary tool was the generalised additive model (GAM), a form of multiple regression. GAMs test for non-linear multidimensional effects using ‘smooth’ terms, which can revert back to simple linear terms (identical to those in linear regression) where the simpler effect gives the best fit to the data [38, 39]. GAMs are particularly useful in nutrition research where there is now abundant evidence that nutrient intakes can have non-linear effects on health outcomes (e.g. [4, 16, 40]). We explored three-dimensional effects of nutrient intake. Because GAMs can estimate non-linear effects, qualitative interpretation of the sign of estimated effects comes through visualisation (rather than a single regression coefficient). Here effects were visualised using the nutrient intake surfaces common to the GFN (Fig. 1). Importantly, GAMs can be used to correct for factors (e.g. sociodemographic status) that might be expected to confound relationships, in the same way as standard linear regression can be used in epidemiology. For each outcome, we fitted a series of eight models exploring different nutritional predictors and making statistical corrections for different factors. Factors explored were income, education level, age, physical activity, number of comorbidities, sex and current smoking status. Models 1 through 4 explored the effects of macronutrient intake with differing degrees of correction. Models 5 and 6 explored the 3-way effects of different combinations of micronutrients, while models 7 and 8 contained terms for both macronutrients and micro-nutrients simultaneously (see Additional file 1, Text S1 for a full description of all models) [41–46].

The main text contains a complete case analysis comprising 3569 observations from 1560 people. In Additional file 1 (Texts S2 and S3) we report sensitivity analyses, where we have imputed missing income data, and also analysed a more exclusive subset of the complete cases dataset (see Additional file 1: Table S1 for population summaries; the exclusive dataset excluded diabetics, individuals on prescribed diets, BMI <22 or >29.9, or with substantial weight fluctuation). These sensitivity analyses estimated similar effects to those in the main text. However, for the exclusive dataset, in many places, these effects (although qualitatively similar) do not meet the criteria for statistical significance. We interpret this latter point as evidence that the effects in the two datasets are similar, but that the power of the complete case analysis is required to detect statistical significance.

### Dietary macronutrients

**Fig. 2:**
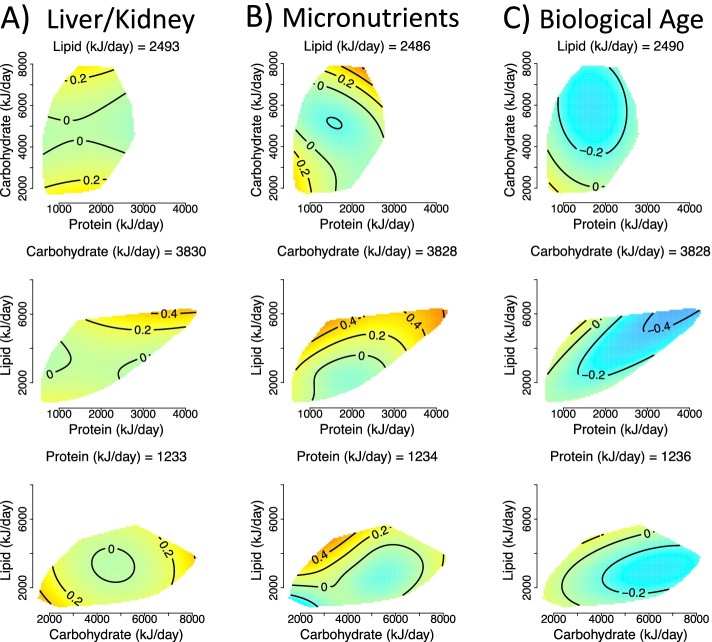
Effects of total dietary intake (kJ/day) of protein, carbohydrates, and fats on **A** liver/kidney function dysregulation (GAM three-way smooth term: edf=9, Ref. df=9, *F*=2.66, *p*<0.01, Dev. Expl.=1.5%, *n*=1834), **B** micronutrient dysregulation (GAM three-way smooth term: edf=14.6, Ref. df=18.1, F=1.8, *p*<0.05, Dev. Expl.=2.21%, *n*=1750) and **C** biological age score as predicted by model 1 (GAM three-way smooth term: edf=9, Ref. df=9, *F*=4.06, *p*<0.001, Dev. Expl.=2.79%, *n*=1796). Surfaces across the top row show effects of protein (*x*-axis), and carbohydrate (*y*-axis) intake, those across the middle row protein and lipid, and the bottom row is carbohydrate and lipid. The third macronutrient is held at the values given on all panels (population median). Warm colours indicate high dysregulation, and cool colours low dysregulation. All scores were *Z*-transformed to one SD, and surfaces colours are scaled such that deep blue and red represent values of at least −0.8 and 0.8 (conventionally considered an effect of large biological magnitude [46])

Our first model, model 1, tested for the effects of macronutrient intake (in kJ/day) on outcomes without any statistical corrections (see Additional file 1: Text S1 for parameters of all models). Because this model does not make correction for any confounders its output shows the unadjusted association between macronutrient intake and dysregulation/ageing scores within the data. We detected statistically significant effects of macronutrient intake on liver/kidney and micronutrient system dysregulation scores, as well as biological age (Fig. 2A–C; see Additional file 1: Table S2 for all statistical model output). Model 1 predicted a relatively minor effect of protein on liver/kidney function dysregulation, and non-linear effects of all carbohydrates and fats on liver/kidney function dysregulation. Individuals who consumed high (> 6000 kJ/day) or low (< 3000 kJ/day) levels of carbohydrates typically had slightly elevated (around 0.25 SD above the population mean) dysregulation scores (Fig. 2A). Very high intakes of lipids (> 4000 kJ/day; note this is within 2SD above the mean lipid intake) had the highest liver/kidney function dysregulation (~0.4 SD above the mean; Fig. 2A). With regard to micronutrient dysregulation scores, individuals with moderately high carbohydrate (5000 to 6000 kJ/day) and low lipid (< 2000 kJ/day) intake had minimal dysregulation. Again, high lipid intake was associated with maximal dysregulation (Fig. 2B). Subjects with protein intake of around 2000 kJ/day were predicted by model 1 to have low dysregulation (Fig. 2B). Biological age was predicted to be the lowest for individuals with high levels of intake for all three macronutrients (Fig. 2C); note that in this analysis nutrient intakes are not expressed relative to any measure of individual requirements.

**Fig. 3:**
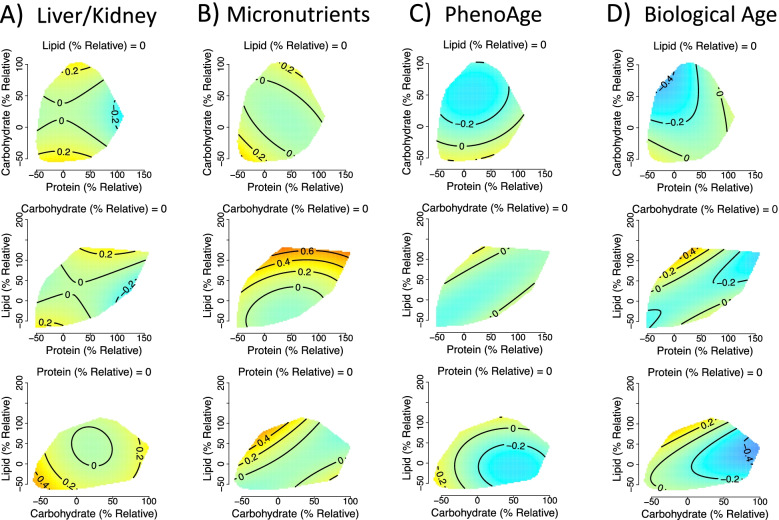
Effects of relative dietary macronutrient intake (relative to the required intake based on age, weight, height, sex and physical activity level; see Additional file 1: Text S4) on **A** liver/kidney function dysregulation, (GAM three-way smooth term: edf=9, Ref. df=9, F=3.8, p<0.001, Dev. Expl.=1.8%, n=1834), **B** micronutrient dysregulation, (GAM three-way smooth term: edf=9, Ref. df=9, F=2, p<0.05, Dev. Expl.=0.9%, n=1750), **C** PhenoAge (GAM three-way smooth term: edf=9, Ref. df=9, F=2, p<0.05, Dev. Expl.=0.9%, n=1834) and **D** biological age (GAM three-way smooth term: edf=9, Ref. df=9, F=2, p<0.05, Dev. Expl.=0.9%, n=1796) score as predicted by model 2. Surfaces across the top row show effects of protein (x-axis), and carbohydrate (y-axis) intake, those across the middle row protein and lipid, and the bottom row is carbohydrate and lipid. The third macronutrient is held at the values given on all panels. Warm colours indicate high dysregulation, and cool colours low dysregulation. All scores were *Z*-transformed to one SD, and surface colours are scaled such that deep blue and red represent values of at least −0.8 and 0.8 (conventionally considered an effect of large biological magnitude [46]). Individuals with a relative intake value of 100, eat 100% more of that macronutrient per day (in kJ) than is predicted to be typical for the population given their age, sex, weight, height and level of physical activity level. Conversely, individuals with a relative intake value of 0 would eat the required amount of that macronutrient per day

Because model 1 shows unadjusted associations that may be confounded, in model 2 we considered dysregulation score as a function of macronutrient intake relative to what we define as the typical energy intake given height, weight, age, sex and physical activity (Additional file 1: Text S4). We again detected effects of macronutrient intake on liver/kidney function and micronutrient dysregulation and biological age score, but in addition, effects on PhenoAge were detected (Fig. 3A–D; Additional file 1: Table S3). Again, this analysis indicated high protein intake relative to what is typical (100% above average) had low liver/kidney function dysregulation scores (Fig. 3A). Interestingly, both PhenoAge and biological age were predicted to be low at elevated carbohydrate levels and typical levels of lipid and protein (Fig. 3C, D). These effects remained after making corrections for potential confounders, both excluding and including the number of comorbidities (models 3 and 4; Additional file 1: Tables S4 and S5), and showed a similar trend within the exclusive dataset (see Additional File 1: Text S3 and Figure S3).

### Dietary micronutrients and nutrient subclasses

**Fig. 4:**
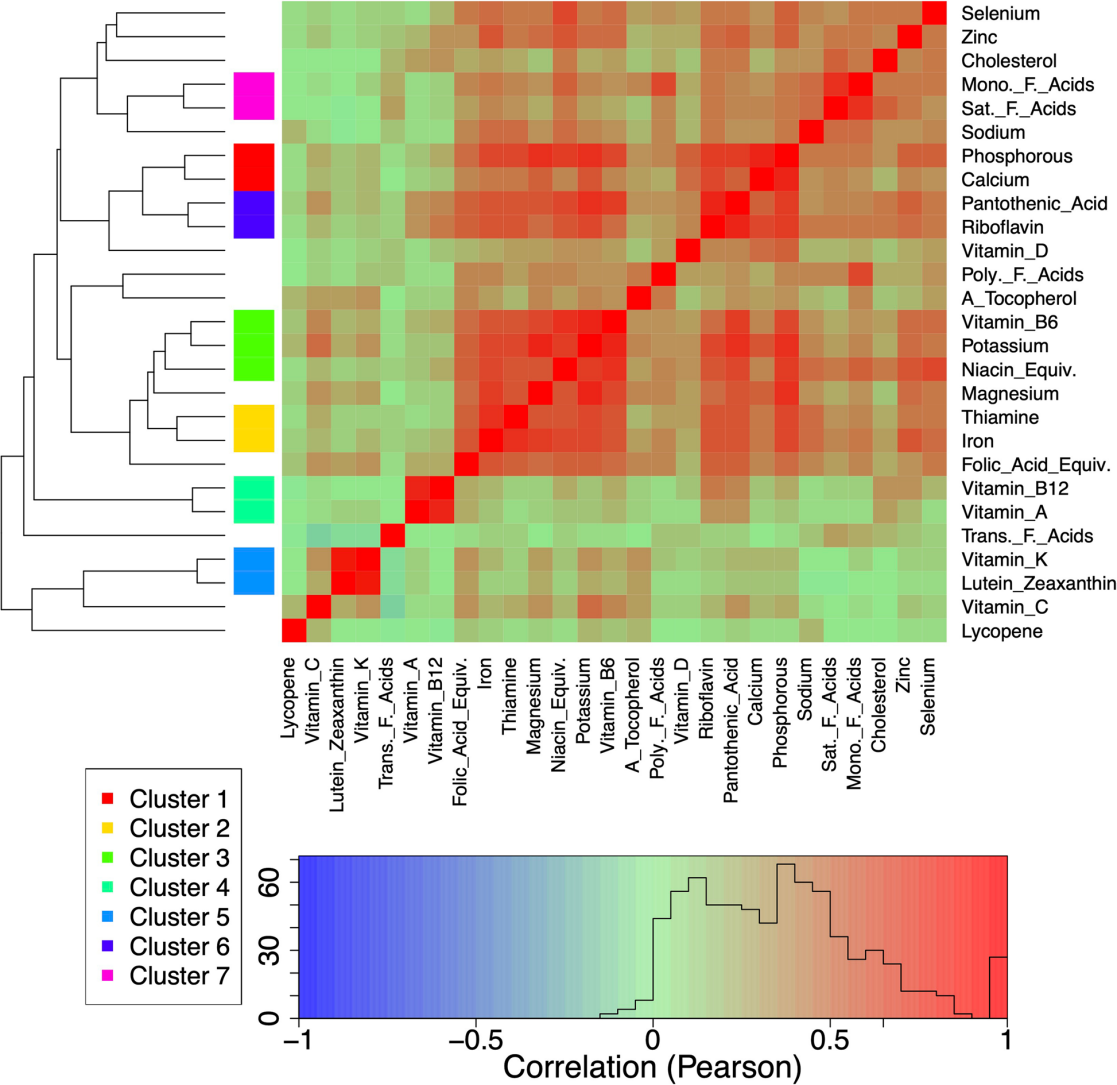
Correlogram of the strength of correlations (Pearson’s correlation) between intakes of micronutrients (*n*=3569). Correlations have been clustered hierarchically based on correlation distance (dendrogram). On the basis of this clustering, we grouped micronutrients with highly correlated intakes (> 0.65) for subsequent dimension reduction using principle components analysis

Intakes of a number of the micronutrient and nutrient subclasses (hereafter referred to collectively as micronutrients) explored were strongly correlated (Fig. 4). Hierarchical clustering based on correlation distance suggested seven clusters of micronutrients with very highly correlated intakes (*r* > 0.65). The first principal component (PC1) of intakes within each of the seven clusters explained between 90 and 100% of the variation of intake of micronutrients within that cluster (Additional file 1: Table S6). PC1 estimates uniformly displayed positive correlations with intakes of micronutrients within the clusters. For subsequent analyses, cluster-specific PC1 values were used as measures of intakes of micronutrients within clusters.

**Fig. 5:**
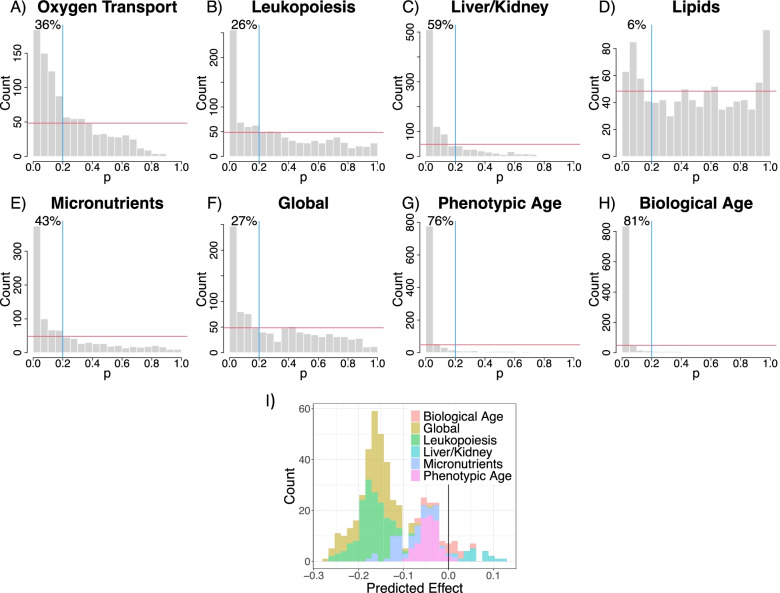
Frequency histograms for the p-values for smooth terms for the 969 unique three-way combinations of micronutrient intakes as given by GAMs (micronutrient-specific models; see Additional file 1: Text S1) where **A** oxygen transport (*n*=3332), **B** leukopoiesis (*n*=3334), **C** liver/kidney function (*n*=1834), **D** lipids (*n*=1991), **E** micronutrients (*n*=1750), **F** global dysregulation (*n*=1718), **G** PhenoAge (*n*=1834) and **H** biological age (*n*=1796) score was treated as an outcome. The red horizontal line indicates the expected frequency under the null hypothesis (that the outcome is unaffected by micronutrient intake) and the blue vertical line demarks *p*=0.2. The percentage of *p*-values falling into the upper left quadrant is given. **I** Frequency histogram of the effects of an increase in *α*-tocopherol intake of 2 SD from the population average as predicted by different models. Predictions come from all models containing significant three-way micronutrient smooth terms involving *α*-tocopherol and make adjustments as per model 6. Predictions assume population average values for all other intakes (including alcohol), income, education level, age, physical activity level (PASE), number of comorbidities, men and non-smoker

After using PCA to reduce dimensionality, we were left with 19 minimally correlated micronutrient/subclass variables (PC1 of clusters or individual micronutrients), making it feasible to run GAMs for all 969 3-way micronutrient combinations for each dysregulation score (micronutrient-specific models; see Additional file 1: Text S1). For all scores except lipid dysregulation, there were a greater number of significant micronutrient smooth terms than would be expected under the null hypothesis (Fig. 5A–H). After correction for the false discovery rate (FDR), there were no significant effects of micronutrient intake on oxygen transport or lipid dysregulation scores. There were 905 models that detected significant effects for at least one score, although 363 of these were solely related to ageing scores (i.e. no significant effect on any dysregulation score; Additional file 1: Table S7). There were 17 combinations for which we detected significant effects on all scores (except oxygen transport and lipid dysregulation). Interestingly all 17 models contained *α*-tocopherol (vitamin E) as one of the three micronutrients. Arguably any one of these micronutrient combinations may be of interest and could warrant further investigation in response to *a priori* hypotheses (results from all models can be found at https://cohenaginglab.github.io/micronuage/). Here however we focus on the effects of *α*-tocopherol, vitamin C and trans-fatty acids because this combination had the highest mean per cent deviance explained across all scores.

**Fig. 6:**
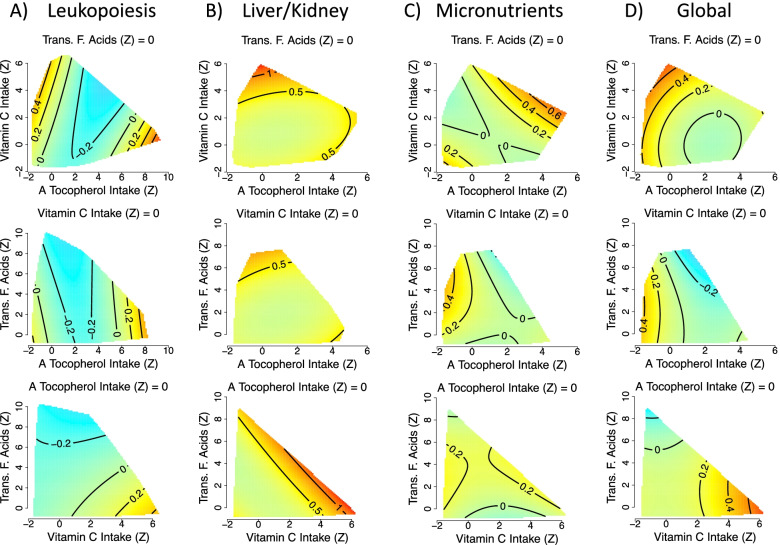
Effects of total dietary micronutrient (*α*-tocopherol, vitamin C and trans-fatty acid intake) intake on **A** leukopoiesis (GAM three-way smooth term: edf=9, Ref. df=9, *F*=3.8, *p*<0.001, Dev. Expl.=6.7%, *n*=3334), **B** liver/kidney function (GAM three-way smooth term: edf=9, Ref. df=9, *F*=1.9, *p*<0.05, Dev. Expl.=3.7%, *n*=1834), **C** micronutrients (GAM three-way smooth term: edf=9, Ref. df=9, *F*=2.3, *p*=0.01, Dev. Expl.=3.8%, *n*=1750) and **D** global (GAM three-way smooth term: edf=9.2, Ref. df=9.5, *F*=2.5, *p*<0.01, Dev. Expl.=7.5%, *n*=1718) dysregulation score as predicted by model 6. Intakes have been *Z*-transformed and are thus in units of SD. In all cases, predictions assume the micronutrient not displayed on either the *x*- or *y*-axis is held at the population mean. Numeric confounding variables included in model 6 were alcohol intake, income, education level, age, physical activity level (PASE) and the number of comorbidities, and predictions assume population mean values. Predictions are for men and assume a non-smoker

In models 5 and 6 we tested for effects of these three micronutrients with correction for confounders, excluding and including comorbidities respectively. In these models, we detected the effects of micronutrients on leukopoiesis, liver/kidney function, micronutrients and global dysregulation (Additional file 1: Tables S8 and S9). The effects varied slightly across the four systems. (Fig. 6A–D for model 6). For example, elevated intakes of trans-fatty acids are predicted to be detrimental for liver/kidney dysregulation, but have a minor beneficial effect on leukopoiesis dysregulation. Nevertheless, consuming around 2SD of *α*-tocopherol above the population mean, while consuming vitamin C and trans-fatty acids at the population mean results in low dysregulation across the four scores, suggesting a systemic benefit of high, but not excessive, *α*-tocopherol intake (Fig. 6). To ensure that increased *α*-tocopherol does not have detrimental interactions with other micronutrients, we screened the complete list of significant micronutrient combinations (after FDR adjustment) for those that included *α*-tocopherol intake; 153 combinations were identified for at least one outcome score. Figure 5I shows the distribution of dysregulation scores (all traits) predicted for a 2SD increase in *α*-tocopherol for any identified effects of micronutrients (after inclusion for potential confounders). For all scores, intakes of *α*-tocopherol 2SD above our sample mean (mean ± SD = 4.75 ± 2.73) is predicted to lead to reductions in dysregulation or ageing *Z*-scores (below the population average) or changes close to 0 (the population average).

### Macro, micro or both

We found statistical support for the effects of both macro and micronutrients on liver/kidney function and micronutrient dysregulation. There can be strong covariances amongst macro and micronutrient intake owing to their co-occurrences in foods (see Additional file 1: Text S5). For example, vitamin C intake is often considered to be a marker of fruit and vegetable consumption [47], and unsurprisingly in this dataset high vitamin C intake coincides with a high carbohydrate diet (Additional file 1: Text S5). One may thus question which of these two nutritional levels is a better predictor of dysregulation, or if both must be considered. We evaluated this by fitting the two three-dimensional effects for macro and micronutrients simultaneously (models 7 and 8, excluding and including comorbidities respectively) and assessing model fit relative to corresponding previous models using the Akaike information criterion (AIC; models with minimal AIC are favoured). For liver/kidney function dysregulation a model including macronutrients is favoured by AIC (Table 2). In contrast, for the micronutrient dysregulation score, a model including only micronutrients is favoured by AIC (Table 2). We also note here that even the most complex models fitted, which include both macro and micronutrients, as well as other potential predictors of health status, explain less than 5% of the deviance in these dysregulation scores for this population (Table 2).

**Table 2:** Akaike information criterion (AIC) and deviance explained (%) by models 4, 6, and 8 for liver/kidney and micronutrient dysregulation. These models contained sex, smoking status, alcohol intake, income, education level, age, physical activity (PASE) and the number of comorbidities, alongside the nutritional predictors stated. See Additional file 1: Table S10 for model variants excluding the number of comorbidities

Dysregulation score	Model number	Nutrient predictors	AIC	Deviance explained
Liver/kidney function	Model 4	Macronutrients	5172	4.1%
	Model 6	Micronutrients	5179	3.69%
	Model 8	Macro + micronutrients	5177	4.82%
Micronutrients	Model 4	Macronutrients	4946	3.63%
	Model 6	Micronutrients	4943	3.81%
	Model 8	Macro + micronutrients	4945	4.66%

## Discussion

Here we used multidimensional modelling techniques to test for associations between nutrient intake and physiological dysregulation in an aged population. We find that consuming above-average protein and *α*-tocopherol intake, which is the most active form of ‘vitamin E’, is associated with lower levels of physiological dysregulation. In contrast, individuals with above-average carbohydrates (given what is typical for height, weight, age sex and physical activity) coupled with typical protein and lipid intakes had minimal measures of biological ageing.

Analysis of the effects of dietary macronutrients on age-specific mortality in mice has shown that protein restriction when coupled with increased carbohydrate intake, extends median lifespan through reduced mortality in middle life, but that higher protein in late life may reduce mortality [21]. Given that our study population consisted entirely of older adults, our findings with regard to physiological dysregulation are consistent with this experimental literature. Our results are also concordant with numerous studies highlighting the need for increased protein intake in older people, in particular, to offset sarcopenia and decreased physical performance associated with ageing (reviewed in [22, 23]). It is interesting to speculate, though, whether reapplying our methods to middle-aged cohorts would detect similar benefits of elevated protein intake; the experimental literature, and some data in humans, suggests perhaps not [20, 21, 48].

Numerous micronutrient interactions have been demonstrated in the experimental absorption literature (e.g. calcium-iron and vitamin C-iron [49]). Our analysis indicates that such interactions may have strong enough effects on health in old age to be detected at the level of the population. For example, elevated vitamin E intake can produce either low or high micronutrient dysregulation scores, depending upon the level of vitamin C intake (Fig. 6C). In vitro and in vivo studies, including a supplementation trial in healthy adults, have detected vitamin C-E absorption interactions, including where simultaneous supplementation leads to elevated circulating levels, and where vitamin C can reduce oxidised vitamin-E recovering its role as an antioxidant [50–52]. Thus, there is a mechanistic basis for what we have detected, and it is certainly worth testing for interactions between these micronutrients in other epidemiological cohorts.

We found that consuming *α*-tocopherol at 2SD above the population average is associated with benefits; in the subject population, this level corresponds to 10.21mg/day of *α*-tocopherol. The World Health Organisation recommended intake of *α*-tocopherol for those aged 65+ is 7.5mg/day in females and 10mg/day in males, thus the value we highlight is not substantially beyond current guidelines [53]. Highlighting the importance of considering non-linearity, more extreme intake patterns (e.g. >4 SDs above average) were associated with harm (Fig. 5). This finding accords with the results of RCTs suggesting excessive vitamin E supplementation may increase all-cause mortality [54]. We also detected a non-linear effect of carbohydrate intake on dysregulation, which suggests individuals at the upper/lower extremes of the observed carbohydrate intakes suffer poor health. Epidemiological and meta-analytic study of the effects of carbohydrates on all-cause mortality in humans has found identical patterns [40], possibly moderated by carbohydrate quality. Generally speaking, our study therefore provides further support to the importance of looking beyond ‘single nutrient at a time’ and monotonic ‘more is better’ analyses [1, 55], to detect the interactive effects of nutrients. For space reasons, we have not presented and discussed the full range of results for all micronutrient intake interactions detected here. However, we have illustrated an approach that can be used to study the effects of multiple micronutrient intakes on health. The GAM-based approach can either be used for discovery (i.e. as means to detect micronutrient interactions) or as a targeted mechanism-driven paradigm to test specific hypotheses about micronutrient interactions derived from experimental biology or nutrition science [49, 56]. We encourage readers to explore http://cohenaginglab.github.io/micronuage where the effects of micronutrient intakes on dysregulation and ageing scores in this population can be visualised interactively, a priori hypotheses explored, and complete models downloaded.

Even in our most complex models, the deviance explained remains at around 5%. This is within the bounds of what analyses of other outcomes in this cohort have found (e.g. [57]). Nevertheless, the surfaces we present here show relatively large effects of nutrition on dysregulation levels, which is potentially important as nutrition is readily modifiable. The low deviance explained may be due to a number of factors including the reporting errors, and systematic reporting biases inherent in epidemiological studies of nutrition [58–60]; the fact we consider additive effects of macro and micronutrients rather than interactions (more complex models are theoretically possible but are limited by sample size and visualisation beyond three-dimensions); and the rarity of the extreme dietary profiles at the edges of the surfaces, where the strongest effects are found (the standard error of the surface is a proxy for sample density; e.g. Additional file 1: Fig. S6). An implication of this latter point is that some of the largest effects of nutrient intake we report will only apply to a small proportion of the population and that our physiology is often robust enough to tolerate relatively wide variation without much consequence. Similar patterns are observed when using the GFN to map evolutionary fitness to organisms that ecologists pre-define as dietary ‘generalists’ [16, 61–63]. This is consistent with an understanding of nutrition in which our ancestors evolved to tolerate an array of dietary patterns [64]. Accordingly, homeostasis can be maintained across a wide array of nutritional states, with the caveat that when diet becomes too extreme dysregulation can increase very rapidly (‘falling off a dietary cliff’). The tolerance for different diets—the size of the plateau in this analogy—could of course vary as a function of genetic or environmental factors that predispose us to greater risk [65].

In contrast to dysregulation, we found that PhenoAge and biological age were minimised on relatively high carbohydrate and lower to moderate protein intakes. Similar results have been observed in numerous experimental studies on the effects of protein to carbohydrate ratio on ageing in model organisms [20]. The somewhat discordant results between dysregulation measures and PhenoAge/Biological Age are not necessarily that surprising. Increasingly, the literature shows that ageing is multivariate and heterogeneous [10, 28]. In this context, our results imply that different dietary patterns come with their own benefits and drawbacks in their effects on the different facets of ageing. A practical consequence of this specificity is that dietary recommendations could be tailored to slow the most advanced ageing processes based on an individual’s biological profile. Given the strength of the apparent trade-off between different ageing/dysregulation scores, it seems unlikely that any one intake pattern can simultaneously minimise all scores. However, once a key target score of interest is identified, multi-criteria optimisation may help define a set of ‘non-dominated’ dietary intakes that maximally improve that score without degrading other outcomes unnecessarily. Regardless of the methods used to advance these findings, replication in other cohorts is needed to confirm the precise patterns we report, and even then, context-specific clinical recommendations will be essential.

An important next step for analyses of this nature will be to complement them with analyses of the effects of food/broad dietary patterns on physiological dysregulation [66]. The intakes of individual nutrients are often markers for broader dietary patterns. High vitamin C/E intake can reflect a diet rich in fresh fruit and vegetables [47], whereas a high trans-fatty acid intake may reflect a diet comprised of processed foods. Such analyses may help to elucidate why, for example, we detect a minor beneficial effect of trans-fatty acids on leukopoiesis dysregulation, where other studies have detected the opposite [67]; it is possible that in this population high trans-fatty acid intakes represent a diet high in dairy, as opposed to processed foods.

## Conclusions

Previously, studies have applied GFN thinking to experimental and observational contexts in humans. They have shown that the macronutrient composition of the diet is associated with biomarkers of cardiovascular health, energy intake, obesity and specific chronic diseases [68–73]. Important as they are, these analyses were concerned with unidimensional measures of health and healthspan. Here, for the first time, we have used the GFN to model holistic measures of systemic functioning during ageing. How results from experimental nutrition and geroscience map onto humans living in the community remains unclear. In part, our understanding has been hampered by a lack of techniques that cut across the complexity inherent to nutrition and biological ageing in real-world contexts; the approach we present here is a promising start. Future applications could include personalised approaches to aid in healthy ageing, and screening of at-risk older adults to ensure they do not fall off the ‘dietary cliff’. Finally, our results advocate against the popular practice of eating to maximise or minimise certain nutrients. The dose-response relationship is often U-shaped, and highly dependent on context (e.g. age, other aspects of diet); targeting in the absence of clear evidence is likely to do more harm than good.

## Methods

### Participant information and dietary intake data

The NuAge Database and Biobank and the present study have been approved by the Research Ethics Board (REB) of the CIUSSS-de-l’Estrie-CHUS (Quebec, Canada; projects 2019-2832 and 2015-868/14-141, respectively). The original sample includes 1587 individuals recruited between November 2003 and June 2005 (T1) to which 206 volunteers were added [34]. They were community-dwelling men and women, aged 67–84 years, able to speak English or French, and in good general health at recruitment. Notably, they had to be free of disabilities in activities of daily living, not cognitively impaired and able to walk 300 metres or to climb 10 stairs without rest. A structured interview was conducted annually in the NuAge Study at baseline (T1) and for the next 3 years (T2, T3 and T4) to gather the following data. Sociodemographic (actual income, education) and lifestyle (smoking, alcohol) information was obtained using a general study questionnaire developed from standard health survey questions [74]. The number of self-reported chronic health conditions (i.e. comorbidities) was computed from an adaptation of the Older Americans Resources and Services (OARS) Multidimensional Functional Assessment questionnaire [75]. Chronic health conditions considered were self-reported cancer (within the last 5 years); hypertension; self-reported liver or gallbladder disease; self-reported surgery of the digestive system; self-reported heart trouble; self-reported circulation trouble in arms or legs; self-reported thrombosis, cerebral haemorrhage, or cerebrovascular accident; self-reported transient cognitive impairment; self-reported Parkinson's disease; self-reported diabetes; self-reported emphysema or chronic bronchitis; self-reported asthma; rheumatoid arthritis, arthritis, or rheumatism; self-reported osteoporosis; self-reported kidney diseases; self-reported thyroid and gland problems; self-reported surgery of the circulatory system; and mini-mental state examination (MMSE) total score between 22 and 26. Usual physical activity was assessed using the Physical Activity Scale for the Elderly (PASE) questionnaire, with higher scores corresponding to higher physical activity levels [76]. Weight and height were measured, and body mass index (BMI = weight [kg]/height [m]^2^) calculated for each participant [34].

Dietary intake data were collected annually (T1 to T4) using 3 non-consecutive 24-h diet recalls [77–79]. Each set included 2 weekdays and 1 weekend day, with the first administered during the annual face-to-face interview and the others by telephone interviews without prior notice. Based on the USDA 5-step multiple-pass method [80], interviewers recorded a detailed description and portion sizes of all foods and beverages consumed by each participant the day before the interview. Only energy and nutrients coming from foods were considered (i.e. excluding supplements). All interviewers were trained registered dietitians. Energy and nutrient intake were computed from the 24-h diet recalls using the CANDAT-Nutrient Calculation System (version 10, ©Godin London Inc.) based on the 2007b version of the Canadian Nutrient File (CNF) from Health Canada and a database of 1200 additional foods that were developed on site [81]. This software produced intakes of the three macronutrients, as well as a full suite of micronutrients and several nutrient subclasses (principally lipid subclasses). We assessed the effects of intakes of all nutrient dimensions available to us.

### Biomarker data and dysregulation score

Biomarkers were chosen based on our previous study [10, 82], their clinical use, and according to their availability in NuAge. Biomarkers were not chosen for their a priori expected relationship to either diet or ageing; indeed, our previous work has shown that dysregulation measures are largely robust to the choice of component markers, reflecting instead an emergent property of the system as a whole. Accordingly, while future work will look at markers of specific interest such as FGF-21, this study does not use markers that are expected individually to integrate metabolism (fatty acids, hydroxybutyrate, insulin, adiponectin, etc.). In total, 30 biomarkers were used to calculate dysregulation globally and for five systems that a previous study validated as being largely independent [10]: (1) oxygen transport; (2) liver/kidney function; (3) leukopoiesis; (4) micronutrients; and (5) lipids (Table 1).

Not all individuals had complete data on biomarkers and nutrition, so sample size depended on the physiological system and the analysis. For the oxygen transport and leukopoiesis systems, the sample size varied between 1224 and 1331. For the other systems, the sample size varied between 654 and 730. The difference in sample size is due to a more extensive serum biomarker analysis conducted in 2016 on a subsample of ~750 individuals that were selected at random amongst the 904 of the 1754 participants who met the following criteria: (1) the individual needed to have blood sampling conducted without any missing intermediate visits; (2) the individual needed at least two visits with blood samples; and (3) there had to be a sufficient number of stored aliquots at all visits to analyse the selected biomarkers.

Dysregulation was calculated using the Mahalanobis distance (D_M_), a statistical distance which measures how deviant or aberrant an individual’s biomarker profile is compared to a reference population. Using D_M_, a global dysregulation score (‘Global’) was calculated with all 30 biomarkers and by the physiological system for each participant at each visit, as previously described [10]. Higher scores indicate higher dysregulation. Age at recruitment in NuAge is restricted from 67 to 84 years, thus, to construct our reference population we used data from the first visit, which represents a population in general good health, according to the inclusion and exclusion criteria (22). Some biomarkers were log- or square-root- transformed as necessary to approach normality. All biomarkers were then centred at the mean of the reference population and divided by the standard deviation of the reference population to standardise them before calculation of dysregulation scores (i.e. UV scaled).

Alongside the 6 measures of physiological dysregulation, we also included two other measures of biological ageing: phenotypic age (PhenoAge) and Klemera-Doubal biological age. PhenoAge measures the predicted age of the individual based on the individual’s mortality risk as assessed via 9 biomarkers and calibrated in NHANES IV. Biological age measures the age of an individual as predicted based on linear projections of a set of age-associated biomarkers. We calculated PhenoAge as described elsewhere [36, 37], although because CRP (C-reactive protein) is not available in the NuAge cohort, we used the mean CRP value from NHANES IV 1999–2010 [37] for all individuals. Validation with individually-imputed values showed a very strong correlation (*r*=0.99) with PhenoAge scores using this mean value; we chose the mean value as the more conservative option and simpler model. PhenoAge showed a weaker correlation with chronological age in our population than previously reported (*r*=0.67 *vs* 0.96 [37];). Exploratory analysis of other datasets in our possession indicated the discrepancy was likely due to the limited age range rather than the absence of CRP (data not shown). Calculation of biological age was based on previous work by Levine [35]. We first searched for biomarkers that correlated with chronological age and found 13 with *r* > 0.1 (*p* ≤ 0.05). After removing three biomarkers with numerous missing values (non-HDL cholesterol, LDL, and estimated glomerular filtration rate), we were left with the following list: haemoglobin, haematocrit, red blood cell count, red cell distribution width, monocyte count, albumin, folate, creatinine, blood urea nitrogen, and lymphocyte percentage. Using these biomarkers, we calculated biological age as previously described [35, 83].

### Analyses

All analyses were performed in the statistical programming environment R [84]. In all cases, dysregulation scores were first log-transformed and then *Z*-transformed (mean centred and divided by one SD) prior to model fitting. Analysis was performed using GAMs [38, 85]. GAMs are a form of the generalised linear (mixed) models (GLMs, and GLMMs) that allow the user to model complex non-linear effects through the inclusion of non-parametric ‘smooth’ terms (usually implemented as a spline). These terms can be specified alongside the conventional linear parametric terms in a linear model. Smooth terms can be additive in a single dimension, or multi-dimensional to test for synergistic interactions. The statistical significance of smooth terms can be interpreted via a p-value, but their effect must be interpreted visually (as opposed to numerically via a regression coefficient). GAMs were implemented using the ‘gam’ function in the R package *mgcv*, and terms estimated by restricted maximum likelihood [38, 39].

We first tested the hypothesis that increased protein is associated with improved health in old age. We estimated the effects of daily intake of macronutrients (protein, carbohydrate and fat, in kJ) on each dysregulation/ageing score using GAMs. In all models, dysregulation/ageing score at an observation was the outcome. In model 1, macronutrient intakes at those observations were fitted as a three-dimensional smooth term (thin-plate spline), and individual subject ID was fitted as a random effect (there are multiple observations per individual). However, the dataset is made up of individuals whose energy requirements likely vary due to height, weight, age, sex and physical activity level. Thus, we also estimated macronutrient intakes relative to what is typical for an individual in this population given the aforementioned variables (estimated from the residuals of a model of intake; see Additional file 1: Text S4). In model 2, we modelled dysregulation scores as a function of relative macronutrient intake using a GAM as above.

In model 3, we tested for the effects of confounders by refitting model 2 to include potential confounders as additive effects; sex (men/women), smoking status (current/not current), current income, number of years of education, alcohol intake (g/day), physical activity level (PASE) and age were included. We ran a separate model with comorbidities as an additional potential confounder (model 4). Numeric predictors were *Z*-transformed prior to fitting and were included in the model as smooth terms, and categorical predictors as parametric terms.

In the second part of our analyses, we considered the effects of those micronutrients for which intake data were available. Micronutrient intakes are likely to be highly correlated. Therefore, we performed hierarchical clustering on correlations (using 1—correlation coefficient) of micronutrient intakes based on correlation distance using the ‘hclust’ function in the *stats* package in base R. For any clusters of highly correlated micronutrients, we performed a principal component analysis (PCA; ‘prcomp’ function in *base* R) and used the first principal component (PC1) as our measure of intake for the nutrients within that cluster. The number of micronutrients adds complexity to understating their interactive effects because (1) the estimation of multidimensional smooth terms in GAMs becomes challenging as the number of dimensions grows and (2) the smooth terms in GAMs must be interpreted visually. For these reasons, we restricted ourselves to considering 3 micronutrient dimensions at a time. For each three-way combination of micronutrient intake (PC1 for clusters) we fitted a GAM with a three-dimensional smooth term for the three micronutrients and a random effect for the individual. We then identified those models with significant effects after correction for the false discovery rate (FDR, *q* < 0.05 [86];). We note that correction by FDR assumes that *p*-values are independent, which is not the case here. However, overlooking this non-independence is conservative in that it will result in fewer significant effects rather than more, and thus we proceeded without correction. In the main text, we interpret specific cases of interest, however, all results and models can be accessed at http://cohenaginglab.github.io/micronuage. We also tested for effects of micronutrients alongside correction for the potential confounders discussed above (models 5 and 6), and by modelling micro and macronutrient intakes simultaneously (models 7 and 8). Note that including two three-dimensional smooth terms (one for micronutrients and one for macronutrients) is not the same as a six-dimensional smooth term; this approach is substantially less power-hungry but still allows us to adjust micronutrient models for macronutrient intake, and vice-versa. See Additional file 1 (Text S1) for a concise list of all models implemented.

Statistical significance was inferred for terms in GAMs based on *α* of 0.05. Where we found it necessary to compare amongst models containing different predictors we used the Akaike Information Criterion (AIC), where smaller values (beyond a margin of two points) indicate a better model fit [44]. Smooth terms from GAMs were interpreted from model predictions. For three-dimensional effects of nutrient intake, we present two-dimensional surface plots, where intakes of nutrients are given on the *x*- and *y*-axes, while the intake of the third nutrient is held constant at a value stated on the figure panel. On all surfaces deep blue/red areas indicate low/high *Z* bio-scores (i.e. good/bad health), respectively.

The main text contains a complete cases analysis where we report results from analyses on all observations for which relevant predictor and dysregulation score data were available (i.e. inclusive dataset with missing income data excluded). Additional file 1, Supplementary Texts S2 and S3 contain the results of two sets of sensitivity analyses. In the first, we have imputed missing income data (31%) as the participant-specific mean value to increase our sample size. In the second we have analysed a subset of the data (<50% of the total data) where we have excluded any observation at which the subject was recorded as either; diabetic (type-1 or 2), reported as being on a medically prescribed diet, having a BMI outside the range of 22 to 29.9, or that came from a subject with a coefficient of variation of weight > 0.04 over the course of all the observations (i.e. for whom weight fluctuated substantially).

Key analyses were performed by two separate members of the team, using broadly the same approach but with no consultation on the detailed analytical decisions (i.e. what might be inferred from seeing the Results but not the Methods). Discrepancies leading to qualitative changes in the conclusions were flagged and resolved. VL performed this validation, with AMS as primary data analyst. Datasets containing all participants’ variables used in this study and those from dietary intakes estimated from 24-h dietary recalls were transmitted by the NuAge team in November–December 2018 and October 2019, respectively. Data for some biomarkers were obtained in a previous secondary project [82].

## Supplementary Information

**Additional file 1. **Contains the following. **Text S1.** Model Descriptions. **Text S2.** Analysis of Inclusive Dataset with Imputation of Missing Income Data. **Text S3.** Analysis of Exclusive Dataset. **Text S4.** Estimation of Relative Intake. **Text S5.** Dietary Macronutrient Composition and Micronutrient Intake. **Table S1.** Variables and Summary Statistics. **Tables S2** though **S5**, **S8** & **S9.** GAM outputs. **Table S6.** Principal Component Analysis. **Table S7.** Models with Significant Effects of Micronutrients. **Table S10** & **S11.** Model AICs. **Figure S1.** & **S3.** Effects of Relative Macronutrient Intake. **Figure S2** & **S4.** Effects of Dietary Micronutrients. **Figure S5.** Effects of Diet Macronutrient Composition on Micronutrient intake. **Figure S6.** Standard Errors for Effects of Macronutrients.

## Data Availability

All data generated during this study are included in this published article, its supplementary information files and publicly available repositories. Code is available at https://github.com/AlistairMcNairSenior/NuAGE_GFN (DOI: 10.5281/zenodo.6984539) All surfaces and associated models from which conclusions are drawn are available at http://cohenaginglab.github.io/micronuage. The raw data are subject to strict restrictions due to ethical concerns and their use must be assessed by the NuAge Research Committee. Requests for access to then raw data should be made to NuAge-crdv@usherbrooke.ca for review.
