# Supplementary material for: Multidimensional associations between nutrient intake and healthy ageing in humans
Source: BMC Biol. 2022 Sep 1;20:196. doi: 10.1186/s12915-022-01395-z (PMC9438070; doi:10.1186/s12915-022-01395-z)
Supplement: Supplementary file 1 — Additional file 1. Contains the following. Text S1. Model Descriptions. Text S2. Analysis of Inclusive Dataset with Imputation of Missing Income Data. Text S3. Analysis of Exclusive Dataset. Text S4. Estimation of Relative Intake. Text S5. Dietary Macronutrient Composition and Micronutrient Intake. Table S1. Variables and Summary Statistics. Tables S2 though S5, S8 & S9. GAM outputs. Table S6. Principal Component Analysis. Table S7. Models with Significant Effects of Micronutrients. Table S10 & S11. Model AICs. Figure S1. & S3. Effects of Relative Macronutrient Intake. Figure S2 & S4. Effects of Dietary Micronutrients. Figure S5. Effects of Diet Macronutrient Composition on Micronutrient intake. Figure S6. Standard Errors for Effects of Macronutrients. [file 12915_2022_1395_MOESM1_ESM.docx]

Additional File 1 for: Multidimensional analysis of diet and healthy aging in humans

# Text S1: Models

For each dysregulation score (*Z*-transformed) we implemented GAMs as follows.

Model 1.

$$y_{ij}=\alpha+f\left( {PkJ}_{i},{CkJ}_{i}, {FkJ}_{i} \right)+ \theta_{j}+\varepsilon_{i},$$

where, *y_ij_* is the *i*th dysregulation score for the *j*th individual, *α* is the intercept value, *f*(*PkJ_i_*, *CkJ_i,_ FkJ_i_*) is a three-dimensional smooth term for the effects of absolute intake of protein, carbohydrate and fat in kilojoules at observation *i*, *θ_j_* is a random effect for the *j*th individual and *ε_i_* is the residual for the *i*th observation; *θ_j_* and *ε_i_* are assumed to be normally distributed with means of 0 and SDs estimated from the data. Because model 1 does not contain any additional covariates, its output shows the unadjusted associations within the data.

Model 2.

$$y_{ij}=\alpha+f\left( P_{i},C_{i}, F_{i} \right)+ \theta_{j}+\varepsilon_{i},$$

which is as in model 1, but the three-dimensional macronutrient smooth term is now relative intake of each macronutrient at the *i*th observation (estimated as described in text S4 ‘Estimation of Relative Intake’).

Model 3.

$$y_{ij}=\alpha+f_{1}\left( P_{i},C_{i}, F_{i} \right)+\sum_{p=2}^{6} f_{p}(x_{pi})+\beta_{1}\times{sex}_{i}+\beta_{2}\times{smoke}_{i}+\theta_{j}+\varepsilon_{i},$$

which is as in model 2 but with additional additive smoothed terms for potential confounding variables, where *f_p_*(*x_p_*) is a smoothing function for the *p*th predictor *x* (*x*_2_ = income, *x*_3_ = alcohol intake, *x*_4_ = age, and *x*_5_ = PASE, and *x*_6_ = number of years of education), at observation i. These numeric confounders were *Z*-transformed prior to model fitting. The model also includes parametric terms (*β*_1_ and *β*_2_) for the effect of sex (0 for men and 1 for women) and current smoking status at observation *i* (0 for non-smoker, 1 for smoker).

Model 4.

$$y_{ij}=\alpha+f_{1}\left( P_{i},C_{i}, F_{i} \right)+\sum_{p=2}^{7} f_{p}(x_{pi})+\beta_{1}\times{sex}_{j}+\beta_{2}\times{smoke}_{i}+\theta_{j}+\varepsilon_{i},$$

which is as in model 3 but includes a further smooth term, *x*_7_, which is number of comorbidities of the individual at observation *i*.

Micronutrient-specific models.

$$y_{ij}=\alpha+f\left( k_{1i},{k_{2}}_{i}, {k_{2}}_{i} \right)+ \theta_{j}+\varepsilon_{i},$$

A model was specified for each unique 3-way combination of micronutrients, where *k*_1_*_i_*, *k*_2_*_i_* and *k*_3_*_i_* are the intakes (*Z*-transformed) of the *k*th combination of 3 micronutrients at the *i*th observation, and other terms are as above.

Model 5.

$$y_{ij}=\alpha+f_{1}\left( k_{1},{k_{2}}_{i}, {k_{3}}_{i} \right)+\sum_{p=2}^{6} f_{p}(x_{pi})+\beta_{1}\times{sex}_{j}+\beta_{2}\times{smoke}_{i}+\theta_{j}+\varepsilon_{i},$$

which is as in model 3 but considers a combination of three micronutrients (or PC1 of clusters thereof), *f*(*k*_1_*_i_*, *k*_2_*_i_*, *k*_3_*_i_*), determined to be of interest following the fitting of micronutrient-specific models.

Model 6.

$$y_{ij}=\alpha+f_{1}\left( k_{1},{k_{2}}_{i}, {k_{3}}_{i} \right)+\sum_{p=2}^{7} f_{p}(x_{pi})+\beta_{1}\times{sex}_{j}+\beta_{2}\times{smoke}_{i}+\theta_{j}+\varepsilon_{i},$$

which is as in model 6 but includes the seventh smoothed term for number of comorbidities (*x*_7_).

Model 7.

$$y_{ij}=\alpha+f_{1}\left( P_{i},C_{i}, F_{i} \right)+\sum_{p=2}^{6} f_{p}(x_{pi})+f_{7}\left( k_{1i},{k_{2}}_{i}, {k_{3}}_{i} \right)+\beta_{1}\times{sex}_{j}+\beta_{2}\times{smoke}_{i}+\theta_{j}+\varepsilon_{i},$$

which is as in model 3 but with *f*_7_(*k*_1_*_i_*, *k*_2_*_i_*, *k*_3_*_i_*), which is an additional three-dimensional smooth term for the intake of a combination of three micronutrients (or PC1 of clusters thereof) determined to be of interest following the fitting of micronutrient-specific models.

Model 8.

$$y_{ij}=\alpha+f_{1}\left( P_{i},C_{i}, F_{i} \right)+\sum_{p=2}^{7} f_{p}(x_{pi})+f_{8}\left( k_{1i},{k_{2}}_{i}, {k_{3}}_{i} \right)+\beta_{1}\times{sex}_{j}+\beta_{2}\times{smoke}_{i}+\theta_{j}+\varepsilon_{i},$$

which is as in model 7 but includes the seventh smoothed term for number of comorbidities (*x*_7_).

# Text S2: Analysis of Inclusive Dataset with Imputation of Missing Income Data

Applying model 1 to the imputed dataset detected significant effects of absolute macronutrient intake on liver/kidney function dysregulation, micronutrient dysregulation and biological age. These are same outcomes as are described in the analysis in the main text, and the estimated effects of nutrition were near identical. Also, as in the main text, we detected effects of relative nutrient intake on liver/kidney function dysregulation, micronutrient dysregulation and PhenoAge and biological age scores in models 2 through 4. Figure S1 is the equivalent to figure 3 in the main text, but with the imputed income data included; the estimated effects are qualitatively identical.

We re-assessed the effects of *α*-tocopherol (vitamin E), vitamin C and trans-fatty acids on dysregulation scores in the imputed dataset using model 6 (i.e. with full correction for socioeconomic factors and comorbidities). As in the main text, we detected effects of this micronutrient combination on leukopoiesis and micronutrient dysregulation (figure S2). We did not detect effects on liver/kidney function or global dysregulation, although in the latter case the smooth term was close to reaching statistical significance (GAM three-way smooth term: edf=9, Ref. df=9, F=1.8, p=0.06, Dev. Expl.=6%). We did however detect effects of micronutrients on lipid system dysregulation (figure S2). These analyses show that lipid dysregulation is low for individuals with relatively high trans-fatty acid and low vitamin C intake; while this is an unexpected observation, this is an extreme dietary profile and the surface in this area is associated with a large degree of sampling error. Nonetheless, as in the main text, elevated levels of *α*-tocopherol (vitamin E) intake to around 2 SD are generally predicted to be beneficial in that they lead to low dysregulation scores (figure S2).

# Text S3: Analysis of Exclusive Dataset

We re-ran our analyses on a subset of the data in which we had excluded any observations where the individual was recorded as any of the following: diabetic (type-1 or 2), reported as being on a medically prescribed diet, having a BMI outside the range of 22 to 29.9, or came from a subject that had a coefficient of variation of weight > 0.04 over the course of all the observations within the dataset. The logic being that, in doing so we would be ensuring that our results were not driven by individuals who had a dietary associated chronic disease and extreme dietary profile that was disproportionately affecting our models. Table S1 shows the profiles of the populations captured within the different datasets. These restrictions excluded over the half of the data, which substantially reduced statistical power. With this reduced dataset our analyses estimated effects with the same sign as those in the main text, but for macronutrients (and liver/kidney function dysregulation with micronutrients) failed to reach the level of statistical significance.

Figure S3 displays the same results as figure 3 in the main text. For this restricted dataset the models estimated the same effects as those for the inclusive dataset, although the effects for liver/kidney function dysregulation, micronutrient dysregulation and biological age score were non-significant. Figure S4 shows the effects of *α*-tocopherol, vitamin C and trans-fatty acid intake on leukopoiesis, liver/kidney function, micronutrient and global dysregulation score. Here all potential confounding variables have been taken into account (i.e., model 6). We see the qualitatively similar effects to those presented in the main text, although the effects are non-significant for all cases but global dysregulation score.

# Text S4: Estimation of Relative Intake

To estimate relative macronutrient intake based on individual requirements, we modelled the mean daily intake of each macronutrient (protein, carbohydrate and fat, in kJ) at an observation, as a function of the subject’s weight, height, age, sex and physical activity level at that observation. Intakes of the three macronutrients were specified as outcomes in a multi-response generalised additive model (GAM), where sex was a categorical parametric predictor, weight, height, and age were fitted as a 3-dimensional smooth term (smoothed by sex), and physical activity level (PASE score) as an additional additive smoothed term. This model estimates the typical intake of each energy-yielding macronutrient as a function of these factors, which are commonly expected to affect energy requirements. From the model we estimated an individual’s relative macronutrient intake as the ratio of their residual for observed intake to that predicted by the model multiplied by 100. Thus, individuals with a relative intake value of 100, eat 100% more of that macronutrient per day (in kJ) than is predicted to be typical for this population given their age, sex, weight, height and level of physical activity. Conversely, individuals with a relative intake value of 0 eat what is the expected given these factors weight, height age, sex and PASE score.

# Text S5: Dietary Macronutrient Composition and Micronutrient Intake

We tested for associations between the composition of the diet in terms of percentage energy from the three macronutrients, protein, carbohydrates and lipids and intake of the micronutrients (or PC1 for clusters) considered. We used mixture models (MMs), also known as Scheffe’s polynomials [41], where micronutrient intake (*z*-transformed to one SD) was the outcome and percentage energy from each macronutrient were the predictors. For each micronutrient we fitted 5 MMs; MM 1 was a null model and MMs 2 through 5 tests for increasingly complex linear through non-linear effects of diet composition on micronutrient intake (see equations 1 through 4 in [41]). It is notable that MM 2 is identical to the substitution models commonly used in nutritional epidemiology [42, 43]. We compared among MMs using Akaike Information Criterion (AIC; [44]), assuming that the simplest model within 2 AIC points of the lowest AIC observed was best supported. MMs were implemented using the *mixexp* package in R [41]. For micronutrients of interest, we visualised the predictions for the AIC-supported MM using right-angle mixture triangles (RMTs; [45]). For all micronutrients AIC favoured models other than the null model (table S11), suggesting that dietary macronutrient composition is associated with intake of micronutrients. Figure S5 shows the association between diet composition and the three micronutrient intakes of interest in the main text as predicted by the AIC favoured MMs; *α*-tocopherol, vitamin C and trans-fatty acids. Diets with a low percentage energy from protein and carbohydrates and high percentage energy from fat had high intake of *α*-tocopherol and trans-fatty acids (figures S5a and S5c). A high carbohydrate diet was associated with high levels of vitamin C, perhaps reflecting intake of fruits and vegetables (figure S5b).

# Supplementary Tables

Table S1: Summary of the variants of the dataset used (see main text). The inclusive dataset comprised 3569 observations (all time points) of 1560 individuals with missing income values excluded and 5152 observations of 1561 individuals including income values imputed. The exclusive dataset comprised 1473 observations of 567 individuals with missing income values excluded. Values shown outside of brackets are the mean and SD of observations excluding observations with missing incomes, while those in brackets correspond to those when imputed incomes are included. For dysregulation scores values are log Mahalanobis distance. For dysregulation and aging scores sample sizes vary among scores with exact values given with corresponding analyses. Note for smoking, the proportion excludes former smokers.

|  | Inclusive Dataset (inc. imputed income) | | Exclusive Dataset | |
| --- | --- | --- | --- | --- |
| Variable (units) | Mean | SD | Mean | SD |
| Age (years) | 76.77 (76.75) | 4.28 (4.30) | 76.68 | 4.29 |
| Income (CAD/yr) | 39514 (39473) | 22211 (22205) | 41716 | 22489 |
| Education (years) | 11.70 (11.71) | 4.52 (4.51) | 12.34 | 4.67 |
| Alcohol Intake (g/d) | 6.61 (6.50) | 11.63 (11.60) | 7.43 | 11.30 |
| Physical activity (PASE score) | 99.70 (97.45) | 51.84 (51.46) | 108.95 | 52.80 |
| Comorbidities (n) | 2.64 (2.59) | 1.66 (1.63) | 2.29 | 1.54 |
| Body weight (kg) | 72.99 (72.74) | 14.24 (14.07) | 69.51 | 9.44 |
| Height (m) | 1.62 (1.62) | 0.09 (0.09) | 1.62 | 0.09 |
| Men (proportion) | 0.5 (0.51) | NA | 1.47 | 0.50 |
| Current Smoker (proportion) | 0.06 (0.06) | NA | 0.04 | 0.20 |
| Protein Intake (kJ/d) | 1241 (1237) | 375 (376) | 1258 | 380 |
| Carbohydrate Intake (kJ/d) | 3879 (3876) | 1138 (1150) | 4017 | 1150 |
| Lipid Intake (kJ/d) | 2566 (2565) | 933 (931) | 2621 | 925 |
| *α*-tocopherol Intake (mg/day) | 4.75 (4.62) | 2.73 (2.73) | 4.75 | 2.76 |
| Vitamin C Intake (mg/day) | 114.8 (115.5) | 66.5 (67.1) | 118.2 | 65.9 |
| Trans-Fatty Acid Intake (g/day) | 0.83 (0.82) | 1.00 (1.00) | 0.84 | 1.01 |
| Oxygen transport Dysregulation (score) | 1.37 (1.40) | 1.02 (1.01) | 1.25 | 1.02 |
| Leukopoiesis Dysregulation (score) | 1.20 (1.08) | 1.05 (1.00) | 1.27 | 1.06 |
| Liver/kidney function Dysregulation (score) | 3.60 (3.60) | 1.00 (1.00) | 3.51 | 0.94 |
| Lipid Dysregulation (score) | 0.80 (0.76) | 0.98 (1.00) | 0.76 | 0.97 |
| Micronutrient Dysregulation (score) | 1.88 (1.90) | 1.02 (1.01) | 1.81 | 1.03 |
| Global Dysregulation (score) | 7.01 (6.91) | 1.03 (0.99) | 6.94 | 0.98 |
| PhenoAge (score) | 73.06 (72.90) | 7.29 (7.16) | 72.30 | 6.90 |
| Biological Age (score) | 76.39 (76.35) | 8.39 (8.40) | 76.12 | 8.33 |

Table S2: Output of model 1 applied to the inclusive data set with missing income data excluded. For parametric terms estimates, standard error (SE), t and p-values are given. For smooth terms, s(…), estimated degrees of freedom (edf), reference degrees of freedom (Ref. df), F and p-values are given. The sample size (n) is the number of observations, and the random effect for subject ID was fitted as the smooth term s(sujetno). The p-values of significant smooth terms for macronutrients are highlighted in bold.

| Dysregulation Score | Parameter | Estimate/edf | SE/Ref. df | t/F | p |
| --- | --- | --- | --- | --- | --- |
| Oxygen transport | Intercept | 0.118 | 0.034 | 3.461 | 0.001 |
| n = 3332: % Dev. Explained = 0.69: AIC = 9456 | s(PROTEIN_kJ.d,CARBO_kJ.d,LIPID_kJ.d) | 9.014 | 9.028 | 0.736 | 0.677 |
|  | s(sujetno) | 0.942 | 1.000 | 16.130 | <0.001 |
| Leukopoiesis | (Intercept) | -0.181 | 0.034 | -5.259 | <0.001 |
| n = 3334: % Dev. Explained = 2.24: AIC = 9438 | s(PROTEIN_kJ.d,CARBO_kJ.d,LIPID_kJ.d) | 20.653 | 27.031 | 0.814 | 0.745 |
|  | s(sujetno) | 0.974 | 1.000 | 36.897 | <0.001 |
| Liver/kidney function | (Intercept) | 0.082 | 0.046 | 1.779 | 0.076 |
| n = 1834: % Dev. Explained = 1.5: AIC = 5200 | s(PROTEIN_kJ.d,CARBO_kJ.d,LIPID_kJ.d) | 9.004 | 9.007 | 2.659 | **0.005** |
|  | s(sujetno) | 0.809 | 1.000 | 4.226 | 0.022 |
| Lipid | (Intercept) | -0.127 | 0.047 | -2.716 | 0.007 |
| n = 1991: % Dev. Explained = 0.98: AIC = 5654 | s(PROTEIN_kJ.d,CARBO_kJ.d,LIPID_kJ.d) | 9.013 | 9.025 | 0.738 | 0.676 |
|  | s(sujetno) | 0.905 | 1.000 | 9.559 | 0.001 |
| Micronutrient | (Intercept) | <0.001 | 0.024 | -0.002 | 0.998 |
| n = 1750: % Dev. Explained = 2.21: AIC = 4962 | s(PROTEIN_kJ.d,CARBO_kJ.d,LIPID_kJ.d) | 14.588 | 18.099 | 1.763 | **0.025** |
|  | s(sujetno) | 0.003 | 1.000 | <0.001 | 0.693 |
| Global | (Intercept) | <0.001 | 0.024 | <0.001 | 1.000 |
| n = 1718: % Dev. Explained = 1.24: AIC = 4889 | s(PROTEIN_kJ.d,CARBO_kJ.d,LIPID_kJ.d) | 13.114 | 15.842 | 0.983 | 0.473 |
|  | s(sujetno) | 0.001 | 1.000 | <0.001 | 0.959 |
| PhenoAge | (Intercept) | -0.151 | 0.049 | -3.096 | 0.002 |
| n = 1834: % Dev. Explained = 1.57: AIC = 5199 | s(PROTEIN_kJ.d,CARBO_kJ.d,LIPID_kJ.d) | 9.007 | 9.015 | 1.711 | 0.081 |
|  | s(sujetno) | 0.925 | 1.000 | 12.399 | <0.001 |
| Biological Age | (Intercept) | -0.152 | 0.049 | -3.082 | **0.002** |
| n = 1796: % Dev. Explained = 2.79: AIC = 5069 | s(PROTEIN_kJ.d,CARBO_kJ.d,LIPID_kJ.d) | 9.010 | 9.020 | 4.060 | <0.001 |
|  | s(sujetno) | 0.925 | 1.000 | 12.260 | <0.001 |

Table S3: Output of model 2 applied to the inclusive data set with missing income data excluded. For parametric terms estimates, standard error (SE), t and p-values are given. For smooth terms, s(…), estimated degrees of freedom (edf), reference degrees of freedom (Ref. df), F and p-values are given. The sample size (n) is the number of observations, and the random effect for subject ID was fitted as the smooth term s(sujetno). The p-values of significant smooth terms for macronutrients are highlighted in bold.

| Dysregulation Score | Parameter | Estimate/edf | SE/Ref. df | t/F | p |
| --- | --- | --- | --- | --- | --- |
| Oxygen transport | Intercept | 0.115 | 0.034 | 3.378 | 0.001 |
| n = 3332: % Dev. Explained = 1.14: AIC = 9454 | s(PROTEIN_kJ.d,CARBO_kJ.d,LIPID_kJ.d) | 13.059 | 15.951 | 1.015 | 0.419 |
|  | s(sujetno) | 0.939 | 1.000 | 15.357 | <0.001 |
| Leukopoiesis | (Intercept) | -0.183 | 0.034 | -5.320 | <0.001 |
| n = 3334: % Dev. Explained = 1.96: AIC = 9446 | s(PROTEIN_kJ.d,CARBO_kJ.d,LIPID_kJ.d) | 17.789 | 23.276 | 0.619 | 0.921 |
|  | s(sujetno) | 0.974 | 1.000 | 37.742 | <0.001 |
| Liver/kidney function | (Intercept) | 0.081 | 0.046 | 1.756 | 0.079 |
| n = 1834: % Dev. Explained = 1.8: AIC = 5194 | s(PROTEIN_kJ.d,CARBO_kJ.d,LIPID_kJ.d) | 9.004 | 9.007 | 3.298 | **0.001** |
|  | s(sujetno) | 0.805 | 1.000 | 4.120 | 0.024 |
| Lipid | (Intercept) | -0.126 | 0.047 | -2.693 | 0.007 |
| n = 1991: % Dev. Explained = 1.08: AIC = 5652 | s(PROTEIN_kJ.d,CARBO_kJ.d,LIPID_kJ.d) | 9.012 | 9.023 | 0.971 | 0.462 |
|  | s(sujetno) | 0.904 | 1.000 | 9.391 | 0.001 |
| Micronutrient | (Intercept) | <0.001 | 0.024 | <0.001 | 1.000 |
| n = 1750: % Dev. Explained = 1.09: AIC = 4968 | s(PROTEIN_kJ.d,CARBO_kJ.d,LIPID_kJ.d) | 9.012 | 9.025 | 2.116 | **0.025** |
|  | s(sujetno) | <0.001 | 1.000 | <0.001 | 0.749 |
| Global | (Intercept) | <0.001 | 0.024 | <0.001 | 1.000 |
| n = 1718: % Dev. Explained = 0.76: AIC = 4887 | s(PROTEIN_kJ.d,CARBO_kJ.d,LIPID_kJ.d) | 9.924 | 10.728 | 1.088 | 0.373 |
|  | s(sujetno) | <0.001 | 1.000 | <0.001 | 0.958 |
| PhenoAge | (Intercept) | -0.140 | 0.049 | -2.888 | 0.004 |
| n = 1834: % Dev. Explained = 1.99: AIC = 5191 | s(PROTEIN_kJ.d,CARBO_kJ.d,LIPID_kJ.d) | 9.003 | 9.006 | 2.601 | **0.006** |
|  | s(sujetno) | 0.915 | 1.000 | 10.804 | 0.001 |
| Biological Age | (Intercept) | -0.150 | 0.050 | -3.028 | 0.003 |
| n = 1796: % Dev. Explained = 1.88: AIC = 5086 | s(PROTEIN_kJ.d,CARBO_kJ.d,LIPID_kJ.d) | 9.002 | 9.005 | 2.198 | **0.020** |
|  | s(sujetno) | 0.922 | 1.000 | 11.826 | <0.001 |

Table S4: Output of model 3 applied to the inclusive data set with missing income data excluded. For parametric terms estimates, standard error (SE), t and p-values are given. For smooth terms, s(…), estimated degrees of freedom (edf), reference degrees of freedom (Ref. df), F and p-values are given. The sample size (n) is the number of observations, and the random effect for subject ID was fitted as the smooth term s(sujetno). The p-values of significant smooth terms for macronutrients are highlighted in bold.

| Dysregulation Score | Parameter | Estimate/edf | SE/Ref. df | t/F | p |
| --- | --- | --- | --- | --- | --- |
| Oxygen transport | Intercept | 0.220 | 0.039 | 5.597 | <0.001 |
| n = 3332: % Dev. Explained = 2.83: AIC = 9406 | sex_male | -0.234 | 0.038 | -6.196 | <0.001 |
|  | smoke_yes | 0.117 | 0.078 | 1.502 | 0.133 |
|  | s(rel_PROTEIN,rel_CARBO,rel_LIPID) | 9.080 | 9.158 | 1.028 | 0.407 |
|  | s(z.alcohol) | 2.501 | 3.128 | 2.599 | 0.046 |
|  | s(z.income) | 1.001 | 1.002 | 2.610 | 0.106 |
|  | s(z.education) | 1.155 | 1.295 | 0.916 | 0.303 |
|  | s(z.age) | 1.002 | 1.003 | 0.716 | 0.397 |
|  | s(z.PASE) | 2.089 | 2.684 | 7.858 | <0.001 |
|  | s(sujetno) | 0.928 | 1.000 | 12.962 | <0.001 |
| Leukopoiesis | Intercept | -0.111 | 0.039 | -2.859 | 0.004 |
| n = 3334: % Dev. Explained = 6.49: AIC = 9313 | sex_male | -0.014 | 0.037 | -0.386 | 0.700 |
|  | smoke_yes | 0.068 | 0.076 | 0.890 | 0.374 |
|  | s(rel_PROTEIN,rel_CARBO,rel_LIPID) | 16.208 | 20.875 | 0.613 | 0.912 |
|  | s(z.alcohol) | 1.001 | 1.003 | 1.563 | 0.211 |
|  | s(z.income) | 1.810 | 2.255 | 8.666 | <0.001 |
|  | s(z.education) | 1.004 | 1.008 | 0.008 | 0.927 |
|  | s(z.age) | 5.909 | 7.095 | 16.554 | <0.001 |
|  | s(z.PASE) | 1.010 | 1.020 | 3.065 | 0.081 |
|  | s(sujetno) | 0.935 | 1.000 | 14.446 | <0.001 |
| Liver/kidney function | Intercept | 0.181 | 0.052 | 3.482 | 0.001 |
| n = 1834: % Dev. Explained = 3.81: AIC = 5176 | sex_male | -0.230 | 0.052 | -4.420 | <0.001 |
|  | smoke_yes | 0.014 | 0.111 | 0.129 | 0.897 |
|  | s(rel_PROTEIN,rel_CARBO,rel_LIPID) | 9.003 | 9.005 | 2.841 | **0.003** |
|  | s(z.alcohol) | 1.631 | 2.029 | 0.578 | 0.592 |
|  | s(z.income) | 1.129 | 1.246 | 2.919 | 0.100 |
|  | s(z.education) | 1.282 | 1.518 | 1.040 | 0.421 |
|  | s(z.age) | 1.466 | 1.804 | 0.896 | 0.302 |
|  | s(z.PASE) | 1.001 | 1.002 | 11.650 | 0.001 |
|  | s(sujetno) | 0.756 | 1.000 | 3.103 | 0.043 |
| Lipid | Intercept | -0.140 | 0.053 | -2.637 | 0.008 |
| n = 1991: % Dev. Explained = 1.78: AIC = 5654 | sex_male | 0.006 | 0.050 | 0.127 | 0.899 |
|  | smoke_yes | 0.285 | 0.108 | 2.638 | 0.008 |
|  | s(rel_PROTEIN,rel_CARBO,rel_LIPID) | 9.021 | 9.042 | 0.883 | 0.542 |
|  | s(z.alcohol) | 1.000 | 1.001 | 1.158 | 0.282 |
|  | s(z.income) | 1.010 | 1.019 | 0.035 | 0.863 |
|  | s(z.education) | 1.001 | 1.002 | 0.042 | 0.840 |
|  | s(z.age) | 1.936 | 2.447 | 0.802 | 0.525 |
|  | s(z.PASE) | 1.002 | 1.004 | 2.060 | 0.152 |
|  | s(sujetno) | 0.892 | 1.000 | 8.263 | 0.002 |
| Micronutrient | Intercept | 0.018 | 0.036 | 0.503 | 0.615 |
| n = 1750: % Dev. Explained = 3.47: AIC = 4944 | sex_male | -0.037 | 0.053 | -0.699 | 0.485 |
|  | smoke_yes | <0.001 | 0.113 | 0.003 | 0.997 |
|  | s(rel_PROTEIN,rel_CARBO,rel_LIPID) | 9.007 | 9.013 | 2.007 | **0.035** |
|  | s(z.alcohol) | 1.000 | 1.000 | 1.688 | 0.194 |
|  | s(z.income) | 1.000 | 1.001 | 0.201 | 0.654 |
|  | s(z.education) | 1.000 | 1.000 | 0.604 | 0.437 |
|  | s(z.age) | 2.586 | 3.269 | 7.300 | <0.001 |
|  | s(z.PASE) | 1.000 | 1.000 | 6.106 | 0.014 |
|  | s(sujetno) | <0.001 | 1.000 | <0.001 | 0.802 |
| Global | Intercept | 0.053 | 0.036 | 1.462 | 0.144 |
| n = 1718: % Dev. Explained = 6.32: AIC = 4809 | sex_male | -0.123 | 0.053 | -2.313 | 0.021 |
|  | smoke_yes | 0.161 | 0.113 | 1.434 | 0.152 |
|  | s(rel_PROTEIN,rel_CARBO,rel_LIPID) | 9.079 | 9.157 | 0.747 | 0.670 |
|  | s(z.alcohol) | 1.001 | 1.001 | 4.323 | 0.038 |
|  | s(z.income) | 1.637 | 2.032 | 1.045 | 0.358 |
|  | s(z.education) | 1.001 | 1.003 | 1.799 | 0.180 |
|  | s(z.age) | 4.103 | 5.113 | 11.626 | <0.001 |
|  | s(z.PASE) | 1.001 | 1.003 | 8.966 | 0.003 |
|  | s(sujetno) | 0.028 | 1.000 | 0.029 | 0.310 |
| PhenoAge | Intercept | 0.285 | 0.026 | 11.044 | <0.001 |
| n = 1834: % Dev. Explained = 52.73: AIC = 3879 | sex_male | -0.617 | 0.037 | -16.895 | <0.001 |
|  | smoke_yes | 0.142 | 0.077 | 1.829 | 0.068 |
|  | s(rel_PROTEIN,rel_CARBO,rel_LIPID) | 9.002 | 9.004 | 3.739 | **<0.001** |
|  | s(z.alcohol) | 1.915 | 2.394 | 3.151 | 0.032 |
|  | s(z.income) | 2.393 | 2.976 | 1.292 | 0.318 |
|  | s(z.education) | 1.000 | 1.000 | 0.902 | 0.342 |
|  | s(z.age) | 1.001 | 1.001 | 1331.371 | <0.001 |
|  | s(z.PASE) | 3.089 | 3.936 | 18.717 | <0.001 |
|  | s(sujetno) | 0.082 | 1.000 | 0.089 | 0.296 |
| Biological Age | Intercept | -0.024 | 0.030 | -0.790 | 0.429 |
| n = 1796: % Dev. Explained = 28.03: AIC = 4549 | sex_male | 0.064 | 0.046 | 1.396 | 0.163 |
|  | smoke_yes | -0.140 | 0.097 | -1.445 | 0.149 |
|  | s(rel_PROTEIN,rel_CARBO,rel_LIPID) | 9.002 | 9.004 | 2.534 | **0.007** |
|  | s(z.alcohol) | 2.030 | 2.535 | 1.753 | 0.136 |
|  | s(z.income) | 1.000 | 1.000 | 1.483 | 0.224 |
|  | s(z.education) | 1.000 | 1.001 | 0.110 | 0.741 |
|  | s(z.age) | 1.800 | 2.272 | 214.337 | <0.001 |
|  | s(z.PASE) | 1.771 | 2.262 | 11.717 | <0.001 |
|  | s(sujetno) | <0.001 | 1.000 | <0.001 | 0.436 |

Table S5: Output of model 4 applied to the inclusive data set with missing income data excluded. For parametric terms estimates, standard error (SE), t and p-values are given. For smooth terms, s(…), estimated degrees of freedom (edf), reference degrees of freedom (Ref. df), F and p-values are given. The sample size (n) is the number of observations, and the random effect for subject ID was fitted as the smooth term s(sujetno). The p-values of significant smooth terms for macronutrients are highlighted in bold.

| Dysregulation Score | Parameter | Estimate/edf | SE/Ref. df | t/F | p |
| --- | --- | --- | --- | --- | --- |
| Oxygen transport | Intercept | 0.216 | 0.039 | 5.552 | <0.001 |
| n = 3332: % Dev. Explained = 3.91: AIC = 9385 | sex_male | -0.255 | 0.038 | -6.761 | <0.001 |
|  | smoke_yes | 0.131 | 0.078 | 1.685 | 0.092 |
|  | s(rel_PROTEIN,rel_CARBO,rel_LIPID) | 14.033 | 17.495 | 0.875 | 0.602 |
|  | s(z.alcohol) | 2.307 | 2.890 | 2.140 | 0.102 |
|  | s(z.income) | 1.002 | 1.004 | 2.084 | 0.149 |
|  | s(z.education) | 1.003 | 1.006 | 0.657 | 0.417 |
|  | s(z.age) | 1.003 | 1.007 | 1.993 | 0.157 |
|  | s(z.PASE) | 1.814 | 2.319 | 5.945 | 0.002 |
|  | s(sujetno) | 0.910 | 1.000 | 10.093 | 0.001 |
|  | s(z.comorb) | 1.003 | 1.006 | 27.717 | <0.001 |
| Leukopoiesis | Intercept | -0.110 | 0.039 | -2.848 | 0.004 |
| n = 3334: % Dev. Explained = 6.51: AIC = 9315 | sex_male | -0.012 | 0.037 | -0.331 | 0.741 |
|  | smoke_yes | 0.067 | 0.076 | 0.871 | 0.384 |
|  | s(rel_PROTEIN,rel_CARBO,rel_LIPID) | 16.330 | 21.060 | 0.634 | 0.907 |
|  | s(z.alcohol) | 1.001 | 1.003 | 1.464 | 0.226 |
|  | s(z.income) | 1.793 | 2.234 | 8.632 | <0.001 |
|  | s(z.education) | 1.004 | 1.009 | 0.014 | 0.905 |
|  | s(z.age) | 5.894 | 7.080 | 16.827 | <0.001 |
|  | s(z.PASE) | 1.008 | 1.015 | 3.258 | 0.072 |
|  | s(sujetno) | 0.934 | 1.000 | 14.031 | <0.001 |
|  | s(z.comorb) | 1.123 | 1.236 | 0.150 | 0.704 |
| Liver/kidney function | Intercept | 0.167 | 0.050 | 3.341 | 0.001 |
| n = 1834: % Dev. Explained = 4.1: AIC = 5172 | sex_male | -0.240 | 0.052 | -4.600 | <0.001 |
|  | smoke_yes | 0.015 | 0.111 | 0.132 | 0.895 |
|  | s(rel_PROTEIN,rel_CARBO,rel_LIPID) | 9.004 | 9.007 | 2.751 | **0.003** |
|  | s(z.alcohol) | 1.119 | 1.229 | 0.193 | 0.659 |
|  | s(z.income) | 1.384 | 1.671 | 2.430 | 0.164 |
|  | s(z.education) | 1.380 | 1.679 | 0.984 | 0.458 |
|  | s(z.age) | 1.631 | 2.042 | 0.844 | 0.411 |
|  | s(z.PASE) | 1.009 | 1.018 | 8.987 | 0.003 |
|  | s(sujetno) | 0.659 | 1.000 | 1.931 | 0.087 |
|  | s(z.comorb) | 1.001 | 1.001 | 6.206 | 0.013 |
| Lipid | Intercept | -0.146 | 0.053 | -2.738 | 0.006 |
| n = 1991: % Dev. Explained = 2.12: AIC = 5652 | sex_male | -0.002 | 0.050 | -0.031 | 0.976 |
|  | smoke_yes | 0.286 | 0.108 | 2.656 | 0.008 |
|  | s(rel_PROTEIN,rel_CARBO,rel_LIPID) | 9.028 | 9.055 | 0.844 | 0.579 |
|  | s(z.alcohol) | 1.000 | 1.001 | 1.543 | 0.214 |
|  | s(z.income) | 1.002 | 1.004 | 0.097 | 0.757 |
|  | s(z.education) | 1.002 | 1.003 | 0.015 | 0.904 |
|  | s(z.age) | 2.061 | 2.607 | 0.817 | 0.529 |
|  | s(z.PASE) | 1.001 | 1.001 | 2.929 | 0.087 |
|  | s(sujetno) | 0.904 | 1.000 | 9.398 | 0.001 |
|  | s(z.comorb) | 1.714 | 2.177 | 2.624 | 0.075 |
| Micronutrient | Intercept | 0.018 | 0.036 | 0.506 | 0.613 |
| n = 1750: % Dev. Explained = 3.63: AIC = 4946 | sex_male | -0.038 | 0.053 | -0.703 | 0.482 |
|  | smoke_yes | 0.001 | 0.113 | 0.012 | 0.990 |
|  | s(rel_PROTEIN,rel_CARBO,rel_LIPID) | 9.007 | 9.014 | 1.987 | **0.037** |
|  | s(z.alcohol) | 1.000 | 1.000 | 1.555 | 0.213 |
|  | s(z.income) | 1.000 | 1.001 | 0.227 | 0.634 |
|  | s(z.education) | 1.000 | 1.000 | 0.632 | 0.427 |
|  | s(z.age) | 2.572 | 3.251 | 7.311 | <0.001 |
|  | s(z.PASE) | 1.000 | 1.000 | 6.165 | 0.013 |
|  | s(sujetno) | <0.001 | 1.000 | <0.001 | 0.808 |
|  | s(z.comorb) | 1.880 | 2.405 | 0.733 | 0.478 |
| Global | Intercept | 0.056 | 0.035 | 1.584 | 0.113 |
| n = 1718: % Dev. Explained = 6.61: AIC = 4806 | sex_male | -0.133 | 0.053 | -2.493 | 0.013 |
|  | smoke_yes | 0.168 | 0.112 | 1.490 | 0.136 |
|  | s(rel_PROTEIN,rel_CARBO,rel_LIPID) | 9.015 | 9.029 | 0.716 | 0.694 |
|  | s(z.alcohol) | 1.001 | 1.001 | 5.001 | 0.025 |
|  | s(z.income) | 1.784 | 2.225 | 1.320 | 0.279 |
|  | s(z.education) | 1.001 | 1.002 | 1.432 | 0.231 |
|  | s(z.age) | 4.129 | 5.144 | 10.987 | <0.001 |
|  | s(z.PASE) | 1.001 | 1.002 | 7.081 | 0.008 |
|  | s(sujetno) | 0.001 | 1.000 | <0.001 | 0.444 |
|  | s(z.comorb) | 1.001 | 1.001 | 4.879 | 0.027 |
| PhenoAge | Intercept | 0.235 | 0.036 | 6.536 | <0.001 |
| n = 1834: % Dev. Explained = 52.55: AIC = 3736 | sex_male | -0.640 | 0.035 | -18.195 | <0.001 |
|  | smoke_yes | 0.141 | 0.075 | 1.887 | 0.059 |
|  | s(rel_PROTEIN,rel_CARBO,rel_LIPID) | 9.004 | 9.008 | 3.260 | **0.001** |
|  | s(z.alcohol) | 1.372 | 1.656 | 1.500 | 0.159 |
|  | s(z.income) | 1.002 | 1.003 | 0.631 | 0.428 |
|  | s(z.education) | 1.000 | 1.000 | 0.831 | 0.362 |
|  | s(z.age) | 1.000 | 1.001 | 1320.810 | <0.001 |
|  | s(z.PASE) | 2.795 | 3.577 | 13.551 | <0.001 |
|  | s(sujetno) | 0.830 | 1.000 | 4.866 | 0.015 |
|  | s(z.comorb) | 7.510 | 8.143 | 19.650 | <0.001 |
| Biological Age | Intercept | -0.051 | 0.042 | -1.209 | 0.227 |
| n = 1796: % Dev. Explained = 30.62: AIC = 4493 | sex_male | 0.039 | 0.045 | 0.857 | 0.391 |
|  | smoke_yes | -0.127 | 0.096 | -1.329 | 0.184 |
|  | s(rel_PROTEIN,rel_CARBO,rel_LIPID) | 9.003 | 9.005 | 2.362 | **0.012** |
|  | s(z.alcohol) | 1.791 | 2.237 | 0.935 | 0.349 |
|  | s(z.income) | 1.000 | 1.000 | 2.600 | 0.107 |
|  | s(z.education) | 1.000 | 1.000 | 0.418 | 0.518 |
|  | s(z.age) | 1.537 | 1.908 | 242.677 | <0.001 |
|  | s(z.PASE) | 1.451 | 1.794 | 8.320 | <0.001 |
|  | s(sujetno) | 0.599 | 1.000 | 1.494 | 0.114 |
|  | s(z.comorb) | 4.117 | 5.103 | 12.555 | <0.001 |

Table S6: Variance statistics from principal component analysis (PCA) of the 7 clusters of micronutrients with highly correlated intakes.

| Cluster | Variance | PC1 | PC2 | PC3 |
| --- | --- | --- | --- | --- |
| 1 | Standard deviation | 497.58 | 160.48 |  |
|  | Proportion of Variance | 0.91 | 0.09 |  |
|  | Cumulative Proportion | 0.91 | 1 |  |
| 2 | Standard deviation | 4.46 | 0.4 |  |
|  | Proportion of Variance | 0.99 | 0.01 |  |
|  | Cumulative Proportion | 0.99 | 1 |  |
| 3 | Standard deviation | 876.56 | 7.43 | 0.28 |
|  | Proportion of Variance | 1 | 0 | 0 |
|  | Cumulative Proportion | 1 | 1 | 1 |
| 4 | Standard deviation | 1329.24 | 3.37 |  |
|  | Proportion of Variance | 1 | 0 |  |
|  | Cumulative Proportion | 1 | 1 |  |
| 5 | Standard deviation | 1752.73 | 45.51 |  |
|  | Proportion of Variance | 1 | 0 |  |
|  | Cumulative Proportion | 1 | 1 |  |
| 6 | Standard deviation | 1.84 | 0.38 |  |
|  | Proportion of Variance | 0.96 | 0.04 |  |
|  | Cumulative Proportion | 0.96 | 1 |  |
| 7 | Standard deviation | 13.59 | 4.58 |  |
|  | Proportion of Variance | 0.9 | 0.1 |  |
|  | Cumulative Proportion | 0.9 | 1 |  |

Table S7: Numbers of models with significant 3-way smooth terms for effects of micronutrients on different combinations of outcomes (micronutrient-specific models); 1 indicates the outcome score is in the group (i.e. 1, 0, 0, 0, 0, 0 indicates leukopoiesis dysregulation alone, where 1, 1, 1, 1, 1, 1 indicates all scores).

| Leukopoiesis | Liver/kidney function | Micronutrient | Global | PhenoAge | Biological Age | Number of Models |
| --- | --- | --- | --- | --- | --- | --- |
| 1 | 0 | 0 | 0 | 0 | 0 | 0 |
| 0 | 1 | 0 | 0 | 0 | 0 | 18 |
| 1 | 1 | 0 | 0 | 0 | 0 | 0 |
| 0 | 0 | 1 | 0 | 0 | 0 | 0 |
| 1 | 0 | 1 | 0 | 0 | 0 | 0 |
| 0 | 1 | 1 | 0 | 0 | 0 | 0 |
| 1 | 1 | 1 | 0 | 0 | 0 | 0 |
| 0 | 0 | 0 | 1 | 0 | 0 | 4 |
| 1 | 0 | 0 | 1 | 0 | 0 | 3 |
| 0 | 1 | 0 | 1 | 0 | 0 | 2 |
| 1 | 1 | 0 | 1 | 0 | 0 | 0 |
| 0 | 0 | 1 | 1 | 0 | 0 | 0 |
| 1 | 0 | 1 | 1 | 0 | 0 | 0 |
| 0 | 1 | 1 | 1 | 0 | 0 | 0 |
| 1 | 1 | 1 | 1 | 0 | 0 | 0 |
| 0 | 0 | 0 | 0 | 1 | 0 | 28 |
| 1 | 0 | 0 | 0 | 1 | 0 | 0 |
| 0 | 1 | 0 | 0 | 1 | 0 | 15 |
| 1 | 1 | 0 | 0 | 1 | 0 | 0 |
| 0 | 0 | 1 | 0 | 1 | 0 | 3 |
| 1 | 0 | 1 | 0 | 1 | 0 | 0 |
| 0 | 1 | 1 | 0 | 1 | 0 | 1 |
| 1 | 1 | 1 | 0 | 1 | 0 | 0 |
| 0 | 0 | 0 | 1 | 1 | 0 | 0 |
| 1 | 0 | 0 | 1 | 1 | 0 | 2 |
| 0 | 1 | 0 | 1 | 1 | 0 | 1 |
| 1 | 1 | 0 | 1 | 1 | 0 | 0 |
| 0 | 0 | 1 | 1 | 1 | 0 | 0 |
| 1 | 0 | 1 | 1 | 1 | 0 | 0 |
| 0 | 1 | 1 | 1 | 1 | 0 | 0 |
| 1 | 1 | 1 | 1 | 1 | 0 | 0 |
| 0 | 0 | 0 | 0 | 0 | 1 | 84 |
| 1 | 0 | 0 | 0 | 0 | 1 | 1 |
| 0 | 1 | 0 | 0 | 0 | 1 | 19 |
| 1 | 1 | 0 | 0 | 0 | 1 | 0 |
| 0 | 0 | 1 | 0 | 0 | 1 | 20 |
| 1 | 0 | 1 | 0 | 0 | 1 | 0 |
| 0 | 1 | 1 | 0 | 0 | 1 | 4 |
| 1 | 1 | 1 | 0 | 0 | 1 | 0 |
| 0 | 0 | 0 | 1 | 0 | 1 | 3 |
| 1 | 0 | 0 | 1 | 0 | 1 | 1 |
| 0 | 1 | 0 | 1 | 0 | 1 | 1 |
| 1 | 1 | 0 | 1 | 0 | 1 | 0 |
| 0 | 0 | 1 | 1 | 0 | 1 | 0 |
| 1 | 0 | 1 | 1 | 0 | 1 | 0 |
| 0 | 1 | 1 | 1 | 0 | 1 | 0 |
| 1 | 1 | 1 | 1 | 0 | 1 | 0 |
| 0 | 0 | 0 | 0 | 1 | 1 | 251 |
| 1 | 0 | 0 | 0 | 1 | 1 | 10 |
| 0 | 1 | 0 | 0 | 1 | 1 | 113 |
| 1 | 1 | 0 | 0 | 1 | 1 | 1 |
| 0 | 0 | 1 | 0 | 1 | 1 | 112 |
| 1 | 0 | 1 | 0 | 1 | 1 | 2 |
| 0 | 1 | 1 | 0 | 1 | 1 | 40 |
| 1 | 1 | 1 | 0 | 1 | 1 | 0 |
| 0 | 0 | 0 | 1 | 1 | 1 | 6 |
| 1 | 0 | 0 | 1 | 1 | 1 | 84 |
| 0 | 1 | 0 | 1 | 1 | 1 | 6 |
| 1 | 1 | 0 | 1 | 1 | 1 | 4 |
| 0 | 0 | 1 | 1 | 1 | 1 | 1 |
| 1 | 0 | 1 | 1 | 1 | 1 | 48 |
| 0 | 1 | 1 | 1 | 1 | 1 | 0 |
| 1 | 1 | 1 | 1 | 1 | 1 | 17 |

Table S8: Output of model 5 applied to the inclusive data set with missing income data excluded. For parametric terms estimates, standard error (SE), t and p-values are given. For smooth terms, s(…), estimated degrees of freedom (edf), reference degrees of freedom (Ref. df), F and p-values are given. The sample size (n) is the number of observations, and the random effect for subject ID was fitted as the smooth term s(sujetno). The p-values of significant smooth terms for micronutrients are highlighted in bold.

| Dysregulation Score | Parameter | Estimate/edf | SE/Ref. df | t/F | p |
| --- | --- | --- | --- | --- | --- |
| Oxygen transport | Intercept | 0.212 | 0.039 | 5.381 | <0.001 |
| n = 3332: % Dev. Explained = 2.8: AIC = 9407 | sex_male | -0.231 | 0.039 | -5.980 | <0.001 |
|  | smoke_yes | 0.115 | 0.078 | 1.489 | 0.137 |
|  | s(z.A_Tocopherol,z.Vitamin_C,z.Trans._F._Acids) | 9.009 | 9.018 | 0.974 | 0.460 |
|  | s(z.alcohol) | 2.423 | 3.033 | 2.817 | 0.037 |
|  | s(z.income) | 1.001 | 1.002 | 2.395 | 0.122 |
|  | s(z.education) | 1.256 | 1.473 | 0.710 | 0.366 |
|  | s(z.age) | 1.001 | 1.002 | 0.953 | 0.329 |
|  | s(z.PASE) | 2.021 | 2.595 | 7.956 | <0.001 |
|  | s(sujetno) | 0.921 | 1.000 | 11.601 | <0.001 |
| Leukopoiesis | Intercept | -0.110 | 0.039 | -2.838 | 0.005 |
| n = 3334: % Dev. Explained = 6.72: AIC = 9281 | sex_male | -0.038 | 0.038 | -1.008 | 0.314 |
|  | smoke_yes | 0.073 | 0.076 | 0.967 | 0.334 |
|  | s(z.A_Tocopherol,z.Vitamin_C,z.Trans._F._Acids) | 9.009 | 9.018 | 3.825 | **<0.001** |
|  | s(z.alcohol) | 1.000 | 1.001 | 2.360 | 0.125 |
|  | s(z.income) | 1.915 | 2.387 | 8.461 | <0.001 |
|  | s(z.education) | 1.002 | 1.004 | 0.001 | 0.981 |
|  | s(z.age) | 5.448 | 6.620 | 15.120 | <0.001 |
|  | s(z.PASE) | 1.027 | 1.054 | 2.743 | 0.101 |
|  | s(sujetno) | 0.946 | 1.000 | 17.430 | <0.001 |
| Liver/kidney function | Intercept | 0.167 | 0.051 | 3.262 | 0.001 |
| n = 1834: % Dev. Explained = 3.39: AIC = 5184 | sex_male | -0.228 | 0.053 | -4.313 | <0.001 |
|  | smoke_yes | 0.045 | 0.111 | 0.407 | 0.684 |
|  | s(z.A_Tocopherol,z.Vitamin_C,z.Trans._F._Acids) | 9.006 | 9.012 | 1.912 | **0.046** |
|  | s(z.alcohol) | 1.821 | 2.277 | 0.526 | 0.726 |
|  | s(z.income) | 1.218 | 1.403 | 2.666 | 0.129 |
|  | s(z.education) | 1.003 | 1.005 | 1.586 | 0.209 |
|  | s(z.age) | 1.626 | 2.036 | 1.099 | 0.319 |
|  | s(z.PASE) | 1.005 | 1.009 | 12.254 | 0.001 |
|  | s(sujetno) | 0.705 | 1.000 | 2.384 | 0.066 |
| Lipid | Intercept | -0.185 | 0.054 | -3.453 | 0.001 |
| n = 1991: % Dev. Explained = 4.78: AIC = 5639 | sex_male | 0.004 | 0.051 | 0.072 | 0.943 |
|  | smoke_yes | 0.304 | 0.107 | 2.844 | 0.005 |
|  | s(z.A_Tocopherol,z.Vitamin_C,z.Trans._F._Acids) | 25.793 | 34.868 | 1.374 | 0.073 |
|  | s(z.alcohol) | 1.001 | 1.003 | 1.461 | 0.227 |
|  | s(z.income) | 1.133 | 1.254 | 0.134 | 0.835 |
|  | s(z.education) | 1.003 | 1.006 | 0.204 | 0.655 |
|  | s(z.age) | 1.577 | 1.965 | 0.354 | 0.671 |
|  | s(z.PASE) | 1.002 | 1.004 | 2.290 | 0.131 |
|  | s(sujetno) | 0.937 | 1.000 | 14.923 | <0.001 |
| Micronutrient | Intercept | <0.001 | 0.036 | -0.011 | 0.991 |
| n = 1750: % Dev. Explained = 3.63: AIC = 4941 | sex_male | <0.001 | 0.054 | -0.005 | 0.996 |
|  | smoke_yes | 0.012 | 0.113 | 0.103 | 0.918 |
|  | s(z.A_Tocopherol,z.Vitamin_C,z.Trans._F._Acids) | 9.013 | 9.027 | 2.344 | **0.013** |
|  | s(z.alcohol) | 1.000 | 1.001 | 3.414 | 0.065 |
|  | s(z.income) | 1.001 | 1.001 | 0.009 | 0.925 |
|  | s(z.education) | 1.001 | 1.001 | 0.013 | 0.910 |
|  | s(z.age) | 2.503 | 3.166 | 6.279 | <0.001 |
|  | s(z.PASE) | 1.001 | 1.002 | 6.349 | 0.012 |
|  | s(sujetno) | <0.001 | 1.000 | <0.001 | 0.955 |
| Global | Intercept | 0.056 | 0.036 | 1.576 | 0.115 |
| n = 1718: % Dev. Explained = 7.26: AIC = 4793 | sex_male | -0.134 | 0.054 | -2.501 | 0.013 |
|  | smoke_yes | 0.185 | 0.112 | 1.662 | 0.097 |
|  | s(z.A_Tocopherol,z.Vitamin_C,z.Trans._F._Acids) | 9.644 | 10.255 | 2.403 | **0.007** |
|  | s(z.alcohol) | 1.000 | 1.001 | 6.684 | 0.010 |
|  | s(z.income) | 1.466 | 1.794 | 0.765 | 0.343 |
|  | s(z.education) | 1.004 | 1.008 | 0.976 | 0.322 |
|  | s(z.age) | 3.879 | 4.848 | 10.016 | <0.001 |
|  | s(z.PASE) | 1.002 | 1.003 | 9.988 | 0.002 |
|  | s(sujetno) | <0.001 | 1.000 | <0.001 | 0.559 |
| PhenoAge | Intercept | 0.268 | 0.029 | 9.106 | <0.001 |
| n = 1834: % Dev. Explained = 52.3: AIC = 3895 | sex_male | -0.599 | 0.037 | -16.083 | <0.001 |
|  | smoke_yes | 0.176 | 0.078 | 2.262 | 0.024 |
|  | s(z.A_Tocopherol,z.Vitamin_C,z.Trans._F._Acids) | 9.004 | 9.008 | 1.866 | 0.053 |
|  | s(z.alcohol) | 1.801 | 2.251 | 1.837 | 0.140 |
|  | s(z.income) | 2.605 | 3.237 | 1.208 | 0.264 |
|  | s(z.education) | 1.000 | 1.000 | 1.042 | 0.308 |
|  | s(z.age) | 1.000 | 1.001 | 1278.023 | <0.001 |
|  | s(z.PASE) | 2.995 | 3.821 | 19.205 | <0.001 |
|  | s(sujetno) | 0.265 | 1.000 | 0.361 | 0.243 |
| Biological Age | Intercept | -0.029 | 0.031 | -0.957 | 0.339 |
| n = 1796: % Dev. Explained = 27.7: AIC = 4559 | sex_male | 0.071 | 0.046 | 1.532 | 0.126 |
|  | smoke_yes | -0.104 | 0.097 | -1.067 | 0.286 |
|  | s(z.A_Tocopherol,z.Vitamin_C,z.Trans._F._Acids) | 9.471 | 9.922 | 1.450 | 0.173 |
|  | s(z.alcohol) | 1.967 | 2.458 | 1.405 | 0.202 |
|  | s(z.income) | 1.001 | 1.002 | 2.368 | 0.124 |
|  | s(z.education) | 1.176 | 1.333 | 0.028 | 0.933 |
|  | s(z.age) | 1.733 | 2.183 | 216.325 | <0.001 |
|  | s(z.PASE) | 1.722 | 2.195 | 12.271 | <0.001 |
|  | s(sujetno) | <0.001 | 1.000 | <0.001 | 0.468 |

Table S9: Output of model 6 applied to the inclusive data set with missing income data excluded. For parametric terms estimates, standard error (SE), t and p-values are given. For smooth terms, s(…), estimated degrees of freedom (edf), reference degrees of freedom (Ref. df), F and p-values are given. The sample size (n) is the number of observations, and the random effect for subject ID was fitted as the smooth term s(sujetno). The p-values of significant smooth terms for micronutrients are highlighted in bold.

| Dysregulation Score | Parameter | Estimate/edf | SE/Ref. df | t/F | p |
| --- | --- | --- | --- | --- | --- |
| Oxygen transport | Intercept | 0.208 | 0.039 | 5.350 | <0.001 |
| n = 3332: % Dev. Explained = 3.51: AIC = 9382 | sex_male | -0.253 | 0.039 | -6.551 | <0.001 |
|  | smoke_yes | 0.125 | 0.077 | 1.623 | 0.105 |
|  | s(z.A_Tocopherol,z.Vitamin_C,z.Trans._F._Acids) | 9.006 | 9.013 | 0.960 | 0.472 |
|  | s(z.alcohol) | 2.282 | 2.860 | 2.333 | 0.080 |
|  | s(z.income) | 1.001 | 1.001 | 2.045 | 0.153 |
|  | s(z.education) | 1.001 | 1.002 | 0.614 | 0.433 |
|  | s(z.age) | 1.001 | 1.002 | 2.324 | 0.127 |
|  | s(z.PASE) | 1.723 | 2.194 | 6.106 | 0.002 |
|  | s(sujetno) | 0.899 | 1.000 | 8.941 | 0.002 |
|  | s(z.comorb) | 1.001 | 1.002 | 26.721 | <0.001 |
| Leukopoiesis | Intercept | -0.110 | 0.039 | -2.828 | 0.005 |
| n = 3334: % Dev. Explained = 6.73: AIC = 9282 | sex_male | -0.036 | 0.038 | -0.944 | 0.345 |
|  | smoke_yes | 0.072 | 0.076 | 0.949 | 0.343 |
|  | s(z.A_Tocopherol,z.Vitamin_C,z.Trans._F._Acids) | 9.009 | 9.019 | 3.840 | **<0.001** |
|  | s(z.alcohol) | 1.000 | 1.001 | 2.219 | 0.136 |
|  | s(z.income) | 1.889 | 2.356 | 8.450 | <0.001 |
|  | s(z.education) | 1.003 | 1.005 | 0.004 | 0.952 |
|  | s(z.age) | 5.432 | 6.604 | 15.190 | <0.001 |
|  | s(z.PASE) | 1.006 | 1.013 | 3.016 | 0.083 |
|  | s(sujetno) | 0.944 | 1.000 | 16.960 | <0.001 |
|  | s(z.comorb) | 1.002 | 1.005 | 0.360 | 0.549 |
| Liver/kidney function | Intercept | 0.152 | 0.048 | 3.140 | 0.002 |
| n = 1834: % Dev. Explained = 3.69: AIC = 5179 | sex_male | -0.238 | 0.053 | -4.499 | <0.001 |
|  | smoke_yes | 0.043 | 0.110 | 0.388 | 0.698 |
|  | s(z.A_Tocopherol,z.Vitamin_C,z.Trans._F._Acids) | 9.002 | 9.004 | 1.935 | **0.043** |
|  | s(z.alcohol) | 1.005 | 1.009 | 0.023 | 0.879 |
|  | s(z.income) | 1.477 | 1.810 | 2.267 | 0.175 |
|  | s(z.education) | 1.003 | 1.006 | 1.485 | 0.224 |
|  | s(z.age) | 1.777 | 2.241 | 0.740 | 0.427 |
|  | s(z.PASE) | 1.013 | 1.026 | 9.404 | 0.002 |
|  | s(sujetno) | 0.567 | 1.000 | 1.307 | 0.129 |
|  | s(z.comorb) | 1.000 | 1.001 | 7.247 | 0.007 |
| Lipid | Intercept | -0.190 | 0.054 | -3.543 | <0.001 |
| n = 1991: % Dev. Explained = 5.13: AIC = 5633 | sex_male | -0.004 | 0.051 | -0.076 | 0.939 |
|  | smoke_yes | 0.304 | 0.107 | 2.847 | 0.005 |
|  | s(z.A_Tocopherol,z.Vitamin_C,z.Trans._F._Acids) | 25.824 | 34.910 | 1.373 | 0.073 |
|  | s(z.alcohol) | 1.001 | 1.002 | 1.880 | 0.170 |
|  | s(z.income) | 1.005 | 1.011 | 0.155 | 0.700 |
|  | s(z.education) | 1.003 | 1.006 | 0.148 | 0.704 |
|  | s(z.age) | 1.724 | 2.168 | 0.483 | 0.670 |
|  | s(z.PASE) | 1.001 | 1.002 | 3.209 | 0.073 |
|  | s(sujetno) | 0.942 | 1.000 | 16.278 | <0.001 |
|  | s(z.comorb) | 1.867 | 2.383 | 2.676 | 0.069 |
| Micronutrient | Intercept | <0.001 | 0.036 | -0.002 | 0.999 |
| n = 1750: % Dev. Explained = 3.81: AIC = 4943 | sex_male | -0.001 | 0.054 | -0.021 | 0.983 |
|  | smoke_yes | 0.013 | 0.113 | 0.114 | 0.909 |
|  | s(z.A_Tocopherol,z.Vitamin_C,z.Trans._F._Acids) | 9.017 | 9.034 | 2.348 | **0.012** |
|  | s(z.alcohol) | 1.000 | 1.001 | 3.214 | 0.073 |
|  | s(z.income) | 1.000 | 1.001 | 0.015 | 0.902 |
|  | s(z.education) | 1.001 | 1.001 | 0.014 | 0.907 |
|  | s(z.age) | 2.486 | 3.145 | 6.291 | <0.001 |
|  | s(z.PASE) | 1.000 | 1.001 | 6.397 | 0.012 |
|  | s(sujetno) | <0.001 | 1.000 | <0.001 | 0.969 |
|  | s(z.comorb) | 1.988 | 2.549 | 0.852 | 0.432 |
| Global | Intercept | 0.060 | 0.036 | 1.700 | 0.089 |
| n = 1718: % Dev. Explained = 7.49: AIC = 4790 | sex_male | -0.144 | 0.054 | -2.672 | 0.008 |
|  | smoke_yes | 0.189 | 0.111 | 1.699 | 0.090 |
|  | s(z.A_Tocopherol,z.Vitamin_C,z.Trans._F._Acids) | 9.245 | 9.485 | 2.483 | **0.007** |
|  | s(z.alcohol) | 1.000 | 1.000 | 7.423 | 0.007 |
|  | s(z.income) | 1.632 | 2.027 | 1.345 | 0.263 |
|  | s(z.education) | 1.001 | 1.002 | 0.726 | 0.394 |
|  | s(z.age) | 3.897 | 4.869 | 9.481 | <0.001 |
|  | s(z.PASE) | 1.001 | 1.001 | 8.047 | 0.005 |
|  | s(sujetno) | <0.001 | 1.000 | <0.001 | 0.730 |
|  | s(z.comorb) | 1.001 | 1.001 | 4.623 | 0.032 |
| PhenoAge | Intercept | 0.223 | 0.036 | 6.124 | <0.001 |
| n = 1834: % Dev. Explained = 56.26: AIC = 3747 | sex_male | -0.623 | 0.036 | -17.395 | <0.001 |
|  | smoke_yes | 0.168 | 0.075 | 2.256 | 0.024 |
|  | s(z.A_Tocopherol,z.Vitamin_C,z.Trans._F._Acids) | 9.002 | 9.004 | 1.798 | 0.064 |
|  | s(z.alcohol) | 1.035 | 1.069 | 0.903 | 0.329 |
|  | s(z.income) | 1.000 | 1.001 | 1.413 | 0.235 |
|  | s(z.education) | 1.000 | 1.000 | 0.939 | 0.333 |
|  | s(z.age) | 1.000 | 1.001 | 1274.454 | <0.001 |
|  | s(z.PASE) | 2.629 | 3.373 | 14.260 | <0.001 |
|  | s(sujetno) | 0.842 | 1.000 | 5.312 | 0.012 |
|  | s(z.comorb) | 7.682 | 8.282 | 20.101 | <0.001 |
| Biological Age | Intercept | -0.051 | 0.042 | -1.213 | 0.226 |
| n = 1796: % Dev. Explained = 30.25: AIC = 4502 | sex_male | 0.045 | 0.046 | 0.981 | 0.327 |
|  | smoke_yes | -0.097 | 0.096 | -1.008 | 0.313 |
|  | s(z.A_Tocopherol,z.Vitamin_C,z.Trans._F._Acids) | 9.049 | 9.099 | 1.364 | 0.203 |
|  | s(z.alcohol) | 1.713 | 2.137 | 0.700 | 0.456 |
|  | s(z.income) | 1.001 | 1.002 | 3.645 | 0.056 |
|  | s(z.education) | 1.001 | 1.003 | 0.135 | 0.715 |
|  | s(z.age) | 1.438 | 1.763 | 256.910 | <0.001 |
|  | s(z.PASE) | 1.375 | 1.673 | 9.102 | <0.001 |
|  | s(sujetno) | 0.552 | 1.000 | 1.233 | 0.135 |
|  | s(z.comorb) | 4.187 | 5.179 | 12.426 | <0.001 |

Table S10: Akaike information criterion (AIC) and deviance explained (%) by models 3, 5, and 7, applied to the inclusive data set with missing income data excluded, for liver/kidney and micronutrients dysregulation. These models contained, alcohol intake, sex, smoking status, income, education level, age, and physical activity (PASE) alongside the nutritional predictors stated.

| Dysregulation Score | Model Number | Nutrient Predictors | AIC | Deviance Explained |
| --- | --- | --- | --- | --- |
| Liver/kidney function | Model 3 | Macronutrients | 5176 | 3.81% |
|  | Model 5 | Micronutrients | 5184 | 3.39% |
|  | Model 7 | Macro + Micronutrients | 5181 | 4.53% |
| Micronutrients | Model 3 | Macronutrients | 4944 | 3.47% |
|  | Model 5 | Micronutrients | 4941 | 3.63% |
|  | Model 7 | Macro + Micronutrients | 4944 | 4.48% |

Table S11. Akaike information criterion (AIC) for mixture models (MM) (see 1) of micronutrient intake (*Z*-transformed) as a function of dietary macronutrient composition (% total energy from protein, carbohydrates and fats; see text S5), as applied to the inclusive data set with missing income data excluded. The models favoured by AIC for each macronutrient is highlighted in bold italics.

| Micronutrient | MM 1 (null) | MM 2 | MM 3 | MM 4 | MM 5 |
| --- | --- | --- | --- | --- | --- |
| Cluster 1 PC1 | 10131 | 10057 | 10044 | 10040 | ***10036*** |
| Cluster 2 PC1 | 10131 | 10120 | ***10096*** | 10098 | 10098 |
| Magnesium | 10131 | 10079 | ***10068*** | 10070 | 10070 |
| Cluster 3 PC1 | 10131 | 10012 | ***10000*** | 10005 | 10001 |
| Sodium | 10131 | 10008 | 9996 | 9987 | ***9985*** |
| Zinc | 10131 | 9890 | ***9870*** | 9875 | 9872 |
| Selenium | 10131 | 9870 | 9862 | 9863 | ***9858*** |
| Cluster 4 PC1 | 10131 | ***10116*** | 10119 | 10123 | 10119 |
| A Tocopherol | 10131 | 10004 | ***10001*** | 10004 | 10000 |
| Lycopene | 10131 | ***10123*** | 10123 | 10129 | 10125 |
| Cluster 5 PC1 | 10131 | ***10088*** | 10088 | 10090 | 10089 |
| Vitamin D | 10131 | ***10067*** | 10069 | 10071 | 10068 |
| Vitamin C | 10131 | 9949 | ***9943*** | 9943 | 9944 |
| Cluster 6 PC1 | 10131 | 10047 | ***10025*** | 10031 | 10027 |
| Folic Acid Equiv. | 10131 | 10090 | ***10084*** | 10087 | 10085 |
| Cholesterol | 10131 | 9429 | 9417 | ***9414*** | 9417 |
| Trans. F. Acids | 10131 | ***9848*** | 9850 | 9855 | 9852 |
| Cluster 7 PC1 | 10131 | 8063 | 8059 | ***8056*** | 8057 |
| Poly. F. Acids | 10131 | 9163 | 9157 | ***9155*** | 9155 |

# Supplementary Figures


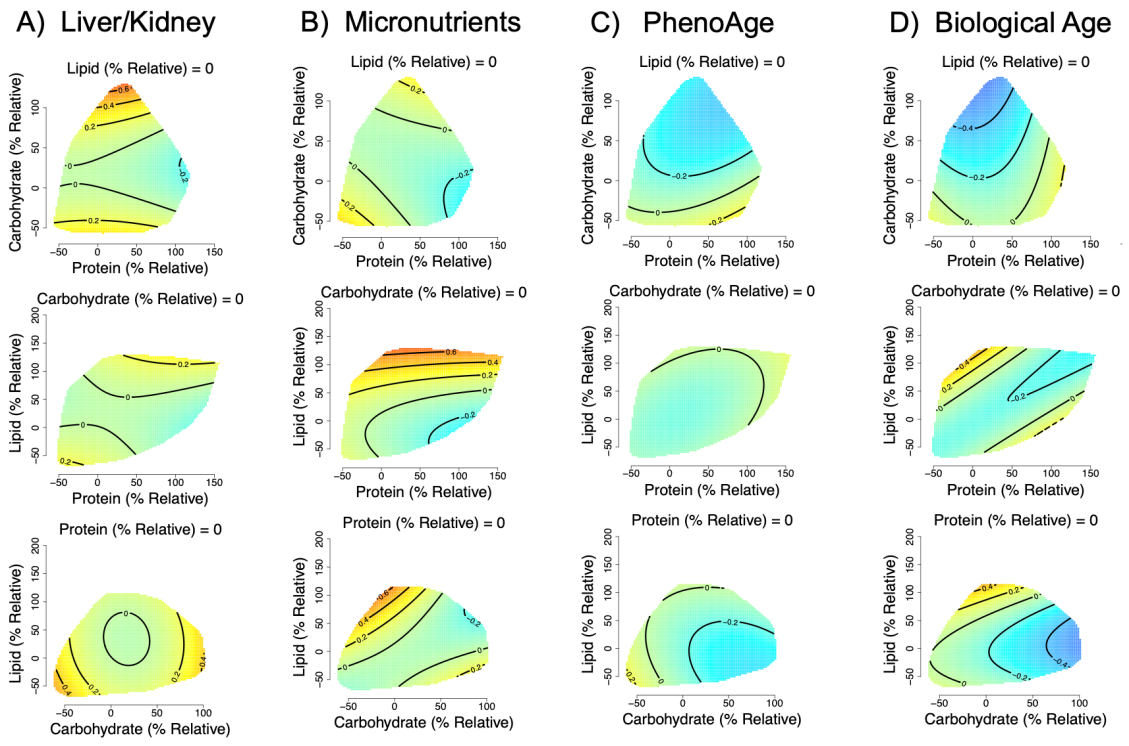


Figure S1. Effects of relative dietary macronutrient intake (relative to the required intake based on age, weight, height sex and physical activity level; see text S1) on A) liver/kidney function dysregulation, (GAM three-way smooth term: edf=9, Ref. df=9, F=4, p<0.001, Dev. Expl.=1.5%, n=2656), B) micronutrient dysregulation, (GAM three-way smooth term: edf=9.2, Ref. df=9.4, F=2.4, p=0.01, Dev. Expl.=0.9%, n=2527), C) PhenoAge (GAM three-way smooth term: edf=9, Ref. df=9, F=2.8, p<0.01, Dev. Expl.=1.6%, n=2656) and D) biological age (GAM three-way smooth term: edf=9, Ref. df=9, F=2.9, p<0.01, Dev. Expl.=1.8%, n=2592) score as predicted by model 2 applied to the inclusive dataset with imputation of missing income data. Surfaces across the top row show effects of protein (x-axis), and carbohydrate (y-axis) intake, those across the middle row protein and lipid, and the bottom row is carbohydrate and lipid. The third macronutrient is held at the values given on all panels. Warm colours indicate high dysregulation, and cool colours low dysregulation. All scores were Z-transformed to one SD, and surfaces colours are scaled such that deep blue and red represent effects of at least -0.8 and 0.8 (conventionally considered an effect of large biological magnitude (6)). Individuals with a relative intake value of 100, eat 100% more of that macronutrient per day (in kJ) than is predicted to be typical for the population given their age, sex, weight, height and level of physical activity level. Conversely, individuals with a relative intake value of 0 would eat the required amount of that macronutrient per day.


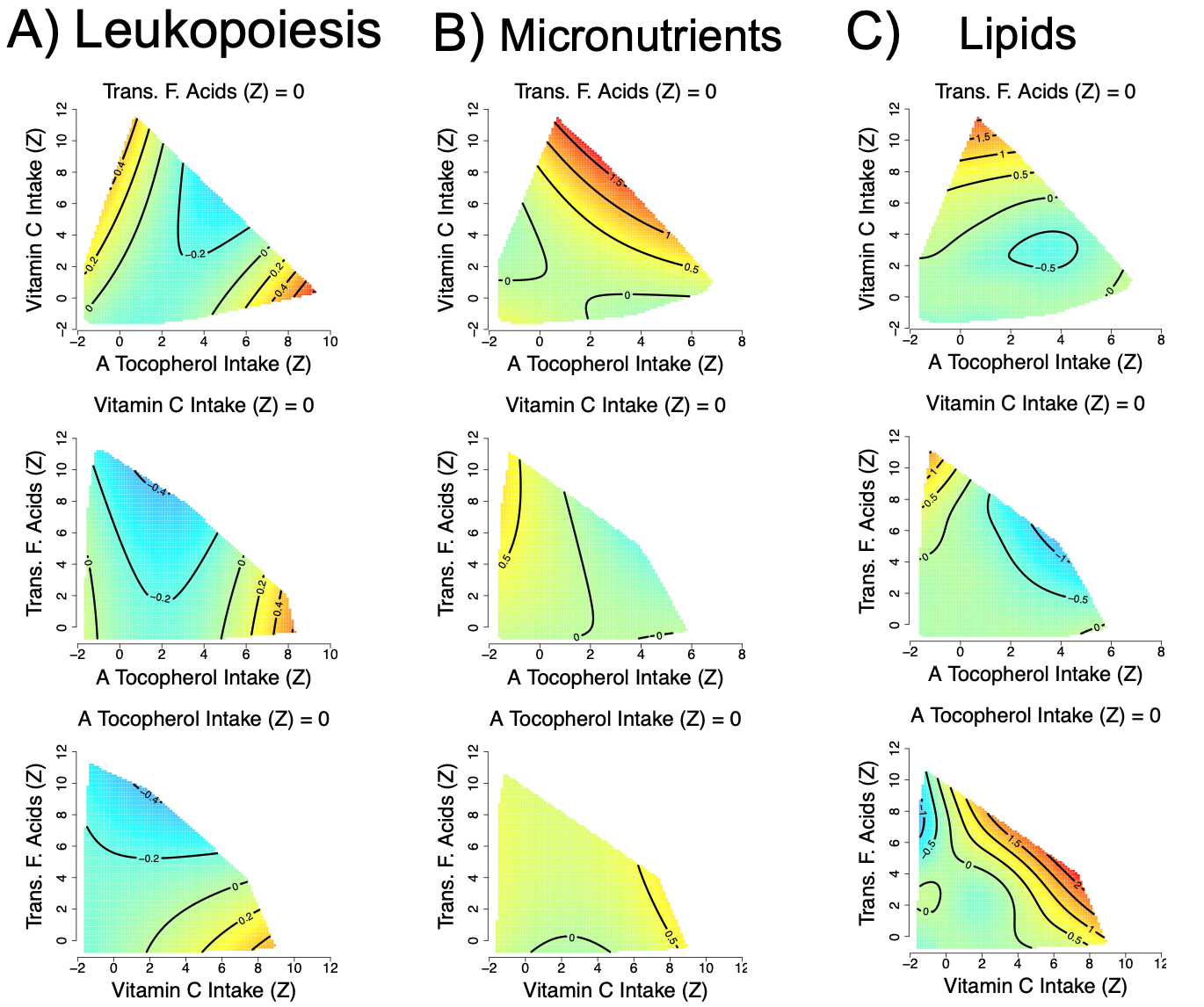


Figure S2. Effects of total dietary micronutrient (*α*-tocopherol, vitamin C and trans-fatty acid intake) intake on A) leukopoiesis (GAM three-way smooth term: edf=9, Ref. df=9, F=2.5, p<0.01, Dev. Expl.=5%, n=4767), B) micronutrient (GAM three-way smooth term: edf=9, Ref. df=9, F=3.6, p<0.001, Dev. Expl.=4%, n=2527) and C) lipid (GAM three-way smooth term: edf=28.7, Ref. df=38.4, F=1.5, p<0.05, Dev. Expl.=5%, n=2805) dysregulation score as predicted by model 6 applied to the inclusive dataset with imputation of missing income data. Intakes have been *Z*-transformed and are thus in units of SD. In all cases predictions assume the micronutrient not displayed on either the x- or y-axis is held at the population mean. Numeric confounding variables included in model 6 were alcohol intake, income, education level, age, physical activity levels (PASE) and number of comorbidities, and predictions assume population mean values. Predictions are for men and assume a non-smoker.


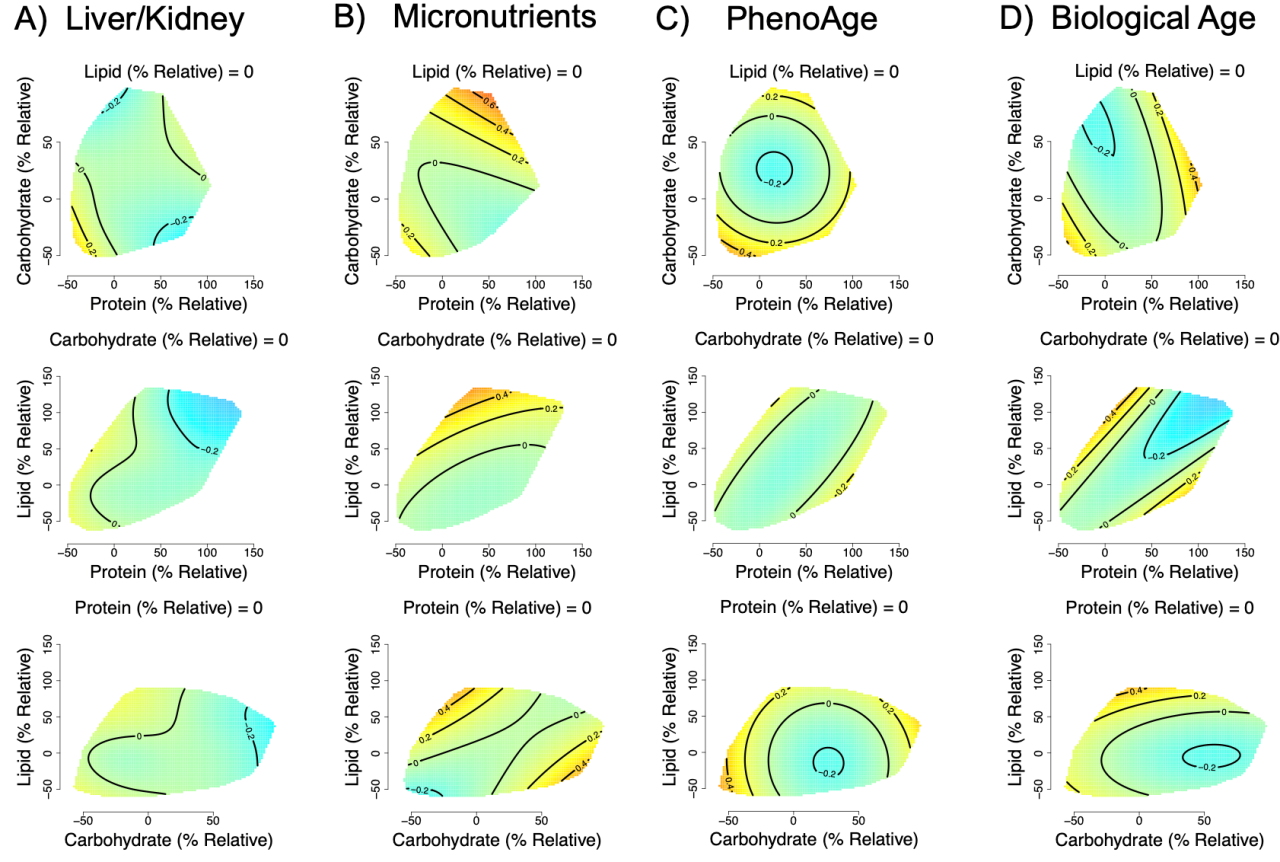


Figure S3. Effects of relative dietary macronutrient intake (relative to typical intake based on age, weight, height sex and physical activity level; see text S1) on A) liver/kidney function (GAM three-way smooth term: edf=13.2, Ref. df=15.9, F=1.1, p=0.34, Dev. Expl.=2.7%, n=860) and B) micronutrient (GAM three-way smooth term: edf=9, Ref. df=9, F=0.9, p=0.57, Dev. Expl.=0.9%, n=825) dysregulation score as predicted by model 1 applied to the exclusive dataset. Effects of relative dietary macronutrient intake (corrected for age, weight, height sex and PASE) on C) PhenoAge (GAM three-way smooth term: edf=9, Ref. df=9, F=2.6, p=0.005, Dev. Expl.=2.6%, n=860) and D) biological age (GAM three-way smooth term: edf=9, Ref. df=9, F=1.7, p=0.08, Dev. Expl.=2%, n=846) dysregulation score as predicted by model 2 applied to the exclusive dataset. Surfaces across the top row show effects of protein (x-axis), and carbohydrate (y-axis) intake, those across the middle row protein and lipid, and the bottom row is carbohydrate and lipid. The third macronutrient is held at the values given on all panels. Warm colours indicate high dysregulation, and cool colours low dysregulation. All scores were Z-transformed to one SD, and surfaces colours are scaled such that, values of -0.8 and 0.8 would be red and blue respectively. Individuals with a relative intake value of 100, eat 100% more of that macronutrient per day (in kJ) than is predicted to be typical for the population given their age, sex, weight, height and level of physical activity level. Conversely, individuals with a relative intake value of 0 would eat the required amount of that macronutrient per day.


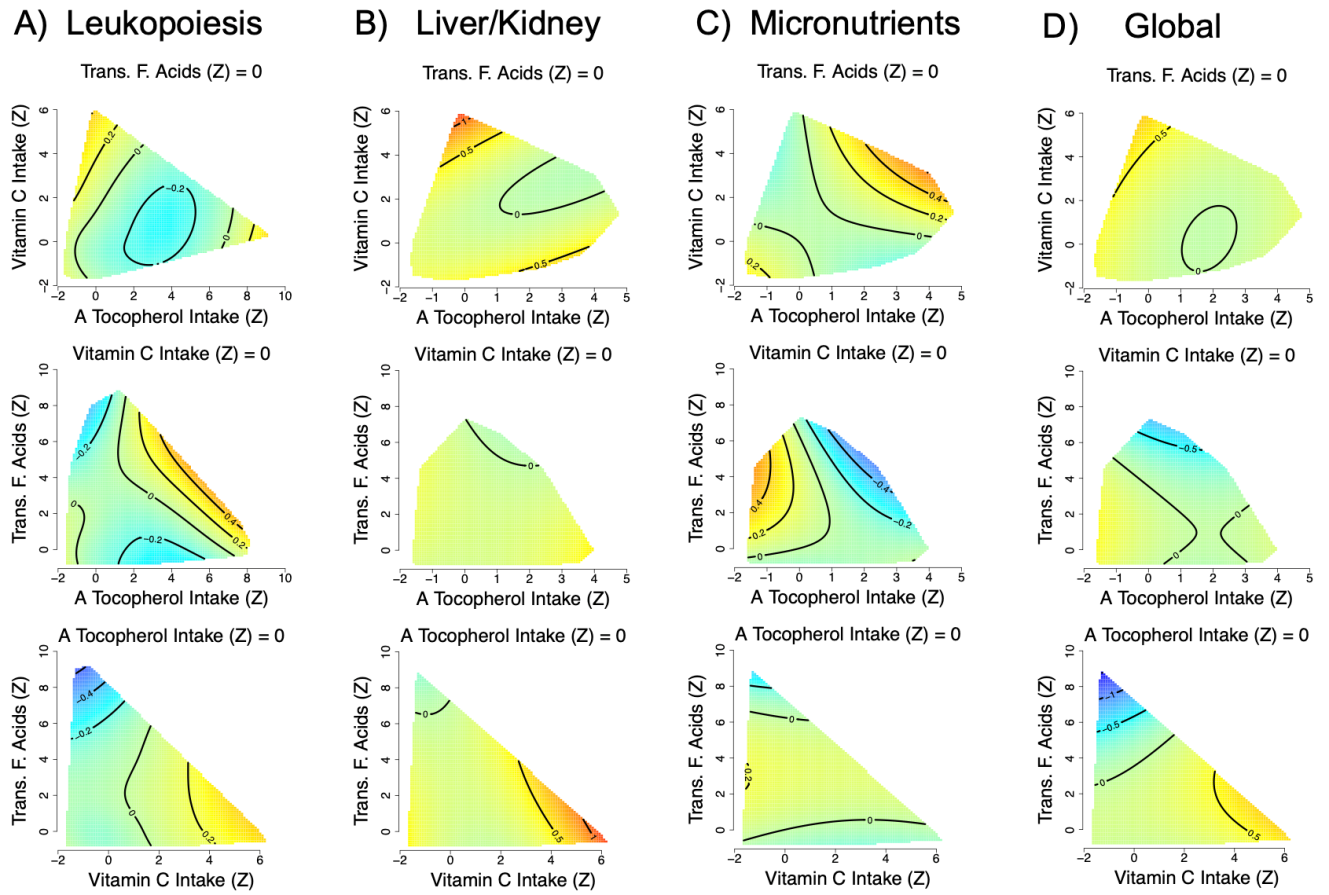


Figure S4. Effects of total dietary micronutrient (*α*-tocopherol, vitamin C and trans-fatty acid intake) intake on A) leukopoiesis (GAM three-way smooth term: edf=12.4, Ref. df=15, F=0.9, p=0.56, Dev. Expl.=8.9%, n=1382), B) liver/kidney function (GAM three-way smooth term: edf=9, Ref. df=9, F=1, p=0.41, Dev. Expl.=5.25%, n=860), C) micronutrient (GAM three-way smooth term: edf=9, Ref. df=9, F=1.03, p=0.41, Dev. Expl.=3.1%, n=825) and D) global (GAM three-way smooth term: edf=9, Ref. df=9, F=1.9, p=0.05, Dev. Expl.=8.25%, n=809) dysregulation score as predicted by model 6 applied to the exclusive dataset. Intakes have been *Z*-transformed and are thus in units of SD. In all cases predictions assume the micronutrient not displayed on either the x- or y-axis is held at the population mean. Numeric confounding variables included in model 6 were alcohol intake, income, education level, age, physical activity level (PASE) and number of comorbidities, and predictions assume population mean values. Predictions are for men and assume a non-smoker.


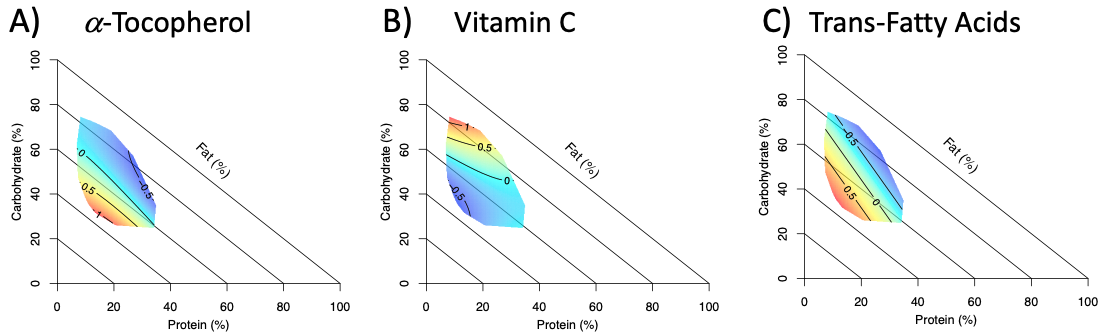


Figure S5. Right angle mixture triangles (5) of associations between dietary macronutrient composition and intake of A) *α*-tocopherol, B) vitamin C and C) trans-fatty acid intake as predicted by AIC favoured mixture models ([41], see text S5, n=3569). Percentage energy from protein and carbohydrates are on the *x*- and *y*-axes respectively, while percentage energy from fats is on the implicit axis. Micronutrient intakes (outcome) have been *z*-transformed such that zero is the population average and units are in one standard deviation.


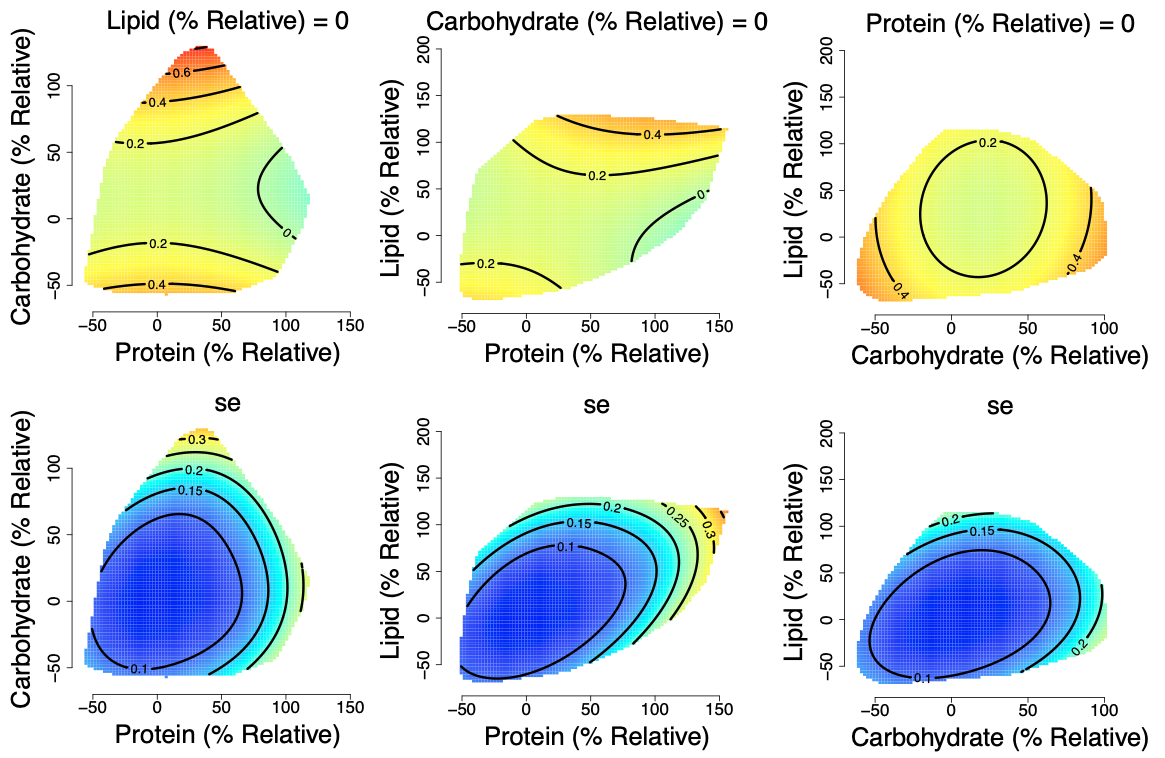


Figure S6. Effects with corresponding standard errors (se) of relative macronutrient intake (relative to estimated requirements; see text S1, n=2656) on liver/kidney function dysregulation score as predicted by model 4 applied to the inclusive dataset with imputation of missing income data. Left hand surfaces show effects of protein (x-axis), and carbohydrate (y-axis) intake, middle show protein and lipid, and the right is carbohydrate and lipid. The third macronutrient is held at the values given on all panels. Warm colours indicate high dysregulation, and cool colours low dysregulation. All scores were Z-transformed to one SD, and surfaces colours are scaled such that deep blue and red represent effects of at least -0.8 and 0.8 (conventionally considered an effect of large biological magnitude [46]). In all cases predictions assume the macronutrient not displayed on either the x- or y-axis is held at the population median. Numeric confounding variables included in model 4 were alcohol intake, income, education level, age, physical activity levels (PASE) and number of comorbidities, and predictions assume population mean values. Predictions are for men and assume a non-smoker.
